# PRMT5 Methylates and Stabilizes EphA2 via Inhibiting Its Ubiquitination and Degradation to Promote Nasopharyngeal Carcinoma Stem Cell Properties

**DOI:** 10.1002/mco2.70697

**Published:** 2026-03-28

**Authors:** Zheng‐Zheng Yu, Xue‐Li Mao, Shan‐Shan Lu, Ruo‐Huang Lu, Wei Zhu, Di Wu, Hong Yi, Wei Huang, Qi Wen, Guo‐Xiang Lin, Ting Zeng, Yun‐Xi Peng, Li Yuan, Ting Ran, Juan Feng, Jinwu Peng, Zhi‐Qiang Xiao

**Affiliations:** ^1^ Department of Pathology Xiangya Hospital Central South University Changsha China; ^2^ Research Center of Carcinogenesis and Targeted Therapy Xiangya Hospital Central South University Changsha China; ^3^ The Higher Educational Key Laboratory for Cancer Proteomics and Translational Medicine of Hunan Province Xiangya Hospital Central South University Changsha China; ^4^ National Clinical Research Center of Geriatric Disorders (Xiangya Hospital) Central South University Changsha China; ^5^ Department of Oral Medicine The Third Xiangya Hospital Central South University Changsha China; ^6^ Department of Nuclear Medicine, The Third Xiangya Hospital Central South University Changsha China; ^7^ Bioland Laboratory (Guangzhou Regenerative Medicine and Health Guangdong Laboratory) Guangzhou China

**Keywords:** cancer stemness, EphA2, nasopharyngeal carcinoma, PRMT5, protein–protein interaction, therapeutic peptide

## Abstract

Both PRMT5 and EphA2 proteins are overexpressed and play a crucial role in multiple cancers, and have been used as targets to develop new anticancer drugs. However, the function and significance of the PRMT5–EphA2 interaction are unclear. Here, we report that PRMT5 bound to EphA2, catalyzed the dimethylation of EphA2 at arginine 816, and then stabilized EphA2 via inhibiting Cbl‐mediated EphA2 ubiquitination and degradation in nasopharyngeal carcinoma (NPC) cells. Functionally, PRMT5 promoted in vitro and in vivo NPC stem cell properties by methylating and stabilizing EphA2. Based on the interacting regions of PRMT5 and EphA2 proteins, we developed a 20 amino acid‐long PRMT5‐derived peptide, P20, which disrupted the connection of PRMT5 with EphA2, degraded EphA2, and suppressed NPC stem cell properties in vitro and in mice. Moreover, the expression levels of PRMT5 and EphA2 in the NPC tissues were significantly higher than those in the normal nasopharyngeal mucosal tissues, and both proteins for predicting the patient's prognosis are superior to individual proteins. Our findings suggest that PRMT5 methylates and stabilizes EphA2 to promote NPC stem cell properties, and the PRMT5‐derived peptide P20 can serve as a novel strategy for targeting EphA2 degradation and inhibiting NPC stem cell properties.

## Graphical abstract

PRMT5 binds and methylates EphA2, which stabilizes EphA2 via inhibiting Cbl‐mediated EphA2 ubiquitination degradation to promote NPC stemness. PRMT5‐derived peptide P20 disrupts PRMT5–EphA2 interaction, which degrades EphA2 via enhancing Cbl‐mediated EphA2 ubiquitination degradation to suppress NPC stemness.

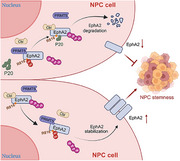

## 1 Introduction

Protein arginine methyltransferase 5 (PRMT5) is a Type II enzyme responsible for the symmetrical dimethylation of protein arginine residues [1]. In addition to epigenetic functions through arginine methylation of histones, PRMT5 can methylate various non‐histone proteins [2, 3, 4]. The importance of arginine methylation in cancer is increasingly recognized [2, 3]. PRMT5 plays a significant role in the initiation and progression of various malignancies [5, 6, 7, 8, 9, 10]. Moreover, PRMT5 is not only important for maintaining pluripotency in both embryonic and neural stem cells [11] but also increases cancer stemness in various types of cancers [12, 13, 14]. As PRMT5 overexpression is associated with the development, progression, and stemness of malignant tumors, inhibitors for targeting PRMT5 enzyme activity have attracted extensive attention [15].

EphA2 (Eph receptor A2), a member of a large family of receptor tyrosine kinases, is overexpressed in various cancers, where it promotes tumor growth and metastasis [16, 17, 18]. Accumulating studies also indicate that EphA2 overexpression promotes the cancer stemness of glioblastomas [19], non‐small cell lung cancer [20], colorectal cancer [21], and head and neck cancer [22, 23]. As EphA2 is an important oncogenic protein associated with cancer stemness, approaches for targeting EphA2 downregulation have been used as anticancer strategies [24, 25].

Our previous study used targeted proteomics to screen proteins interacting with EphA2 in nasopharyngeal carcinoma (NPC) cells and identified PRMT5 as one of the proteins binding with EphA2 [26]. Protein–protein interaction (PPI) regulates various physiological and pathological processes through affecting protein post‐translational modification, stability, and subcellular location. PPI plays a crucial role in cancers, representing a pivotal target for chemical and biological interventions [27, 28]. Preclinical studies have demonstrated that inhibition of PPI by peptides is an efficient anticancer strategy [26, 29, 30].

NPC is one of the most common malignancies in southern China and Southeast Asia and poses a major public health problem in these areas. NPC is characterized by aggressive behaviors, and often invades local tissues and metastasizes to distant sites [31]. Cancer stem cells (CSCs) are a small subset of rare special cancer cells within the tumor cell population, which drive tumor initiation, growth, recurrence, metastasis, and drug resistance, and possess self‐renewal and differentiation capability [32]. Like other cancers, NPC also contains CSCs or cancer stem‐like cells, which promote tumor recurrence, metastasis, and radiochemotherapy resistance [23, 33, 34].

In this work, we found that PRMT5 methylated and stabilized EphA2 via inhibiting its ubiquitination and degradation, which increased NPC stemness, and developed a PRMT5‐derived peptide disrupting PRMT5–EphA2 interaction and targeting EphA2 degradation, which inhibited NPC stemness. Our data reveal the role and mechanism of PRMT5–EphA2 interaction in NPC, and present a potential strategy for treating NPC with a peptide.

## 2 Results

### 2.1 PRMT5 Binds and Methylates EphA2 to Increase Its Stability by the Ubiquitin Proteasome Pathway

**FIGURE 1:**
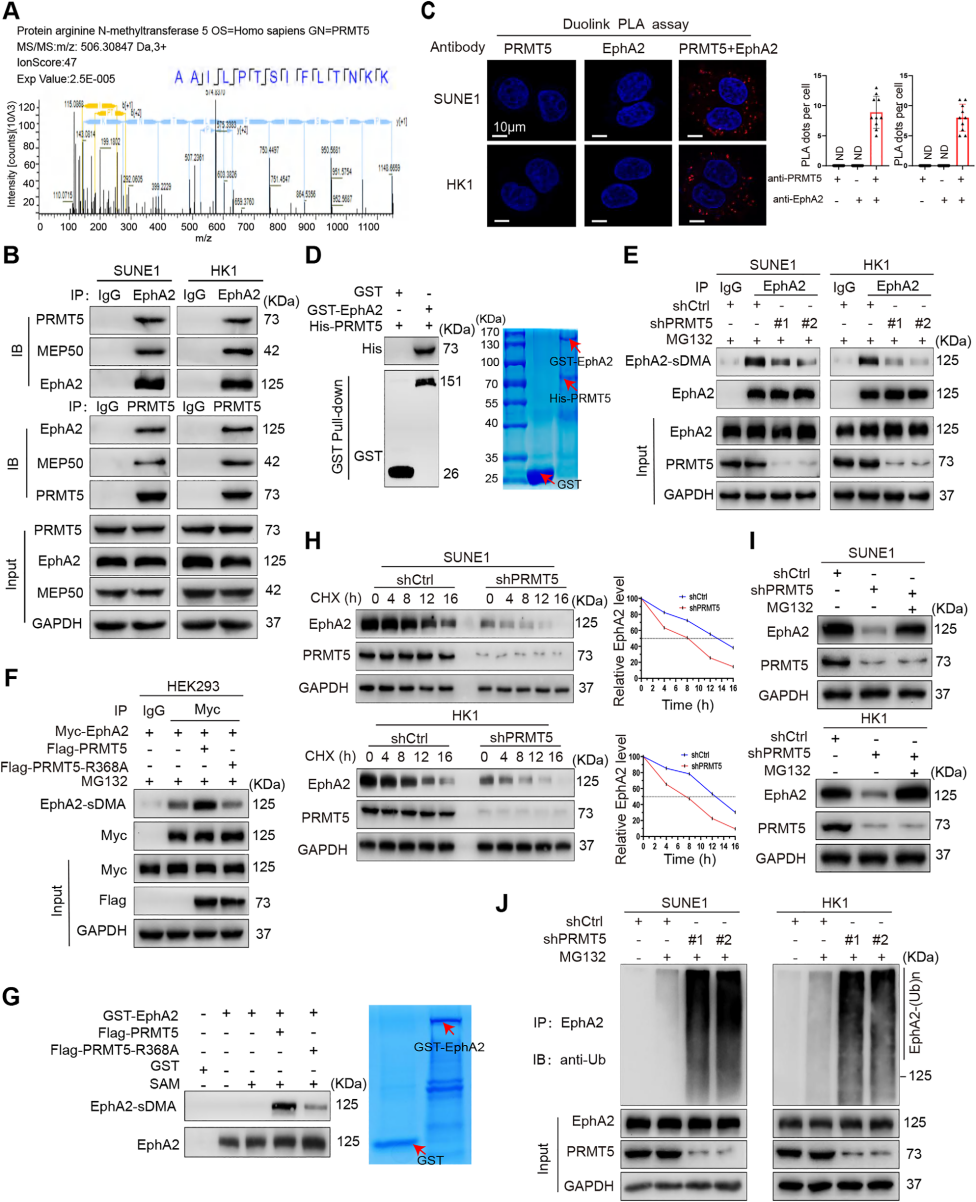
PRMT5 binds and methylates EphA2 to increase its stability via the ubiquitin proteasome pathway. (A) Identification of PRMT5 as a protein interacting with EphA2 by mass spectrometry. The amino acid sequence of a doubly charged peptide with *m*/*z* 506.30847 is identified as AAILPTSIFLTNKK, and Mascot search shows the peptide matched with PRMT5. (B) Co‐IP (co‐immunoprecipitation) showing PRMT5–EphA2 interaction and MEP50 co‐immunoprecipitated with PRMT5 and EphA2 in the SUNE1 and HK1 NPC cells. (C) Duolink in situ proximity ligation assay (PLA) showing PRMT5–EphA2 interaction in the SUNE1 and HK1 NPC cells. Red dots: PLA signal. Scale bars: 10 µm. (D) GST pull‐down showing direct interaction of PRMT5 and EphA2. Purified His‐tagged PRMT5 protein is mixed with purified GST or GST‐tagged EphA2 protein immobilized on glutathione beads. Samples are electrophoresed and immunoblotted with antibodies against GST or His. (E) Co‐IP showing that PRMT5 knockdown decreases EphA2 methylation in the SUNE1 and HK1 NPC cells. (F) Co‐IP showing the effect of wild‐type (WT) PRMT5 and catalytically inactive mutant PRMT5–R368A on EphA2 methylation in the HEK293 cells co‐transfected with the indicated plasmids. (G) In vitro methylation assay showing the effect of WT PRMT5 and PRMT5–R368A on EphA2 methylation. Immunoprecipitated WT PRMT5 or PRMT5–R368A protein is incubated with purified GST‐EphA2 and S‐adenosyl‐l‐methionine (SAM). Samples are electrophoresed and immunoblotted with anti‐symmetric dimethylarginine antibody. (H) Western blot showing the effect of PRMT5 knockdown on EphA2 protein stability in the SUNE1 and HK1 NPC cells treated with 20 µg/mL cycloheximide (CHX) for the indicated times. (I) Western blot showing restoration of EphA2 protein levels by proteasome inhibitor MG132 in the PRMT5 knockdown SUNE1 and HK1 NPC cells. (J) Co‐IP showing that PRMT5 knockdown increases EphA2 polyubiquitination in the SUNE1 and HK1 NPC cells. shPRMT5, PRMT5 knockdown by shRNA; shCtrl, scramble nontarget shRNA; sDMA, symmetric dimethylarginine; GST, glutathione S‐transferase; SAM, S‐adenosyl‐l‐methionine; ND, not detected; Ub, ubiquitin; WT, wild‐type; IP, immunoprecipitation; IB, immunoblotting.

We previously used immunoprecipitation conjugated with mass spectrometry analysis (IP‐MS) to screen proteins interacting with EphA2 in the NPC cells [26], and 1352 proteins, including PRMT5, were identified (Figure 1A). The mass spectrometry data of all identified proteins are available via ProteomeXchange with the dataset identifier PXD015242. To validate PRMT5 binding to EphA2, co‐immunoprecipitation (Co‐IP) was performed to detect PRMT5–EphA2 interaction. The results showed that PRMT5 interacted with EphA2 in the NPC cells (Figure 1B) and in the HEK293 cells with the ectopic expression of PRMT5 and EphA2 (Figure S1A). As PRMT5 functions optimally within a complex containing its regulatory partner methylosome protein 50 (MEP50) [3, 4], we investigated whether MEP50 is present in the PRMT5–EphA2 complex. Co‐IP showed that MEP50 co‐precipitated with PRMT5 and EphA2 (Figure 1B), indicating that MEP50 is involved in PRMT5‐methylating EphA2 in the NPC cells. Duolink in situ proximity ligation assay also showed that PRMT5 interacted with EphA2 in the two NPC cell lines (Figure 1C). GST pull‐down using purified proteins showed that PRMT5 directly interacted with EphA2 (Figure 1D). Collectively, these data demonstrate that PRMT5 interacts with EphA2 in the NPC cells.

Next, we immunoprecipitated EphA2 protein and detected EphA2 methylation by Western blot using anti‐symmetric dimethylarginine (sDMA) antibody. The results showed that PRMT5 knockdown resulted in a significant reduction in EphA2 methylation in the NPC cells (Figure 1E). Moreover, wild‐type (WT) PRMT5 but not its catalytically inactive mutant PRMT5–R368A increased EphA2 methylation in the HEK293 cells (Figure 1F). In vitro methylation assay also showed that PRMT5 catalyzed EphA2 methylation (Figure 1G). These data demonstrate that PRMT5 methylates EphA2 in an enzyme activity‐dependent manner.

As arginine methylation affects protein stabilization [6, 13], we investigated whether PRMT5 has an influence on EphA2 stability. The results showed that EphA2 was rapidly degraded in the NPC cells with PRMT5 knockdown (Figure 1H), although its mRNA level remained unchanged (Figure S1B), indicating that PRMT5 plays a crucial role in stabilizing EphA2 protein. Moreover, the proteasome inhibitor MG132 reversed the decrease of EphA2 protein observed in PRMT5 knockdown NPC cells (Figure 1I), suggesting that PRMT5 knockdown destabilizes EphA2 via a proteasome‐dependent mechanism.

Given that ubiquitination drives EphA2 degradation [35, 36], we determined the capacity of PRMT5 to regulate EphA2 ubiquitination and observed that PRMT5 knockdown promoted EphA2 polyubiquitination in the NPC cells (Figure 1J). Moreover, overexpression of WT PRMT5 but not PRMT5–R368A increased EphA2 protein stability (Figure S1C) and reduced EphA2 polyubiquitination (Figure S1D) in the HEK293 cells. The results indicate that PRMT5 increases EphA2 protein stability by inhibiting its ubiquitination and degradation in an enzyme activity‐dependent manner. Next, we examined whether PRMT5 inhibits the K48‐ or K63‐linked polyubiquitination of EphA2, and observed that PRMT5 efficiently inhibited K48‐linked polyubiquitination, but had no detectable effect on K63‐linked polyubiquitination (Figure S1E). To corroborate the requirement of PRMT5 methyltransferase activity for stabilizing EphA2, NPC cells were treated with PJ‐68, a small molecule inhibitor of PRMT5 [14], and subjected to further analyses. The result showed that PJ‐68 treatment diminished the dimethylation, expression, and stability of EphA2, and increased EphA2 polyubiquitination (Figure S2A–D). Moreover, the decrease of EphA2 protein in the PJ‐68‐treated NPC cells was reversed by treatment with the proteasome inhibitor MG132 (Figure S2E). The results confirm that PRMT5 methyltransferase activity is required for stabilizing EphA2 in NPC cells. Collectively, these data indicate that PRMT5 binds and methylates EphA2 to enhance its stability by ubiquitin–proteasome pathway in NPC cells.

### 2.2 PRMT5‐Catalyzed EphA2 R816 Dimethylation Prevents Cbl‐Mediated EphA2 From Ubiquitination and Degradation

IP‐MS was performed to screen the dimethylated arginines of EphA2 in the NPC cells. A total of five dimethylated arginines (R244, R327, R816, R890, and R907) were identified in the EphA2 (Figure S3A). To investigate which arginine residue is dimethylated by PRMT5, we mutated these arginines into lysines (methylation inactive mutation) in the EphA2, respectively, co‐transfected each EphA2 mutant and PRMT5 into HEK293 cells, followed by co‐IP analysis, and found that only the methylation inactive mutant EphA2–R816K substantially reduced EphA2 methylation (Figure S3B). Consistently, PRMT5 obviously increased the methylation levels of WT EphA2 but not EphA2–R816K in the HEK293 cells (Figure S3C). Next, we investigated whether the R816K mutation affects EphA2 stability, and observed that exogenous EphA2–R816K stability was obviously decreased as compared to exogenous WT EphA2 in the endogenous EphA2 knockdown NPC cells (Figure S3D). Consistently, EphA2–R816K stability was obviously decreased as compared to WT EphA2 in the HEK293 cells co‐transfected with the indicated plasmids (Figure S3E). Collectively, these data suggest that PRMT5 increases EphA2 protein stability by catalyzing dimethylation of EphA2 at R816.

**FIGURE 2:**
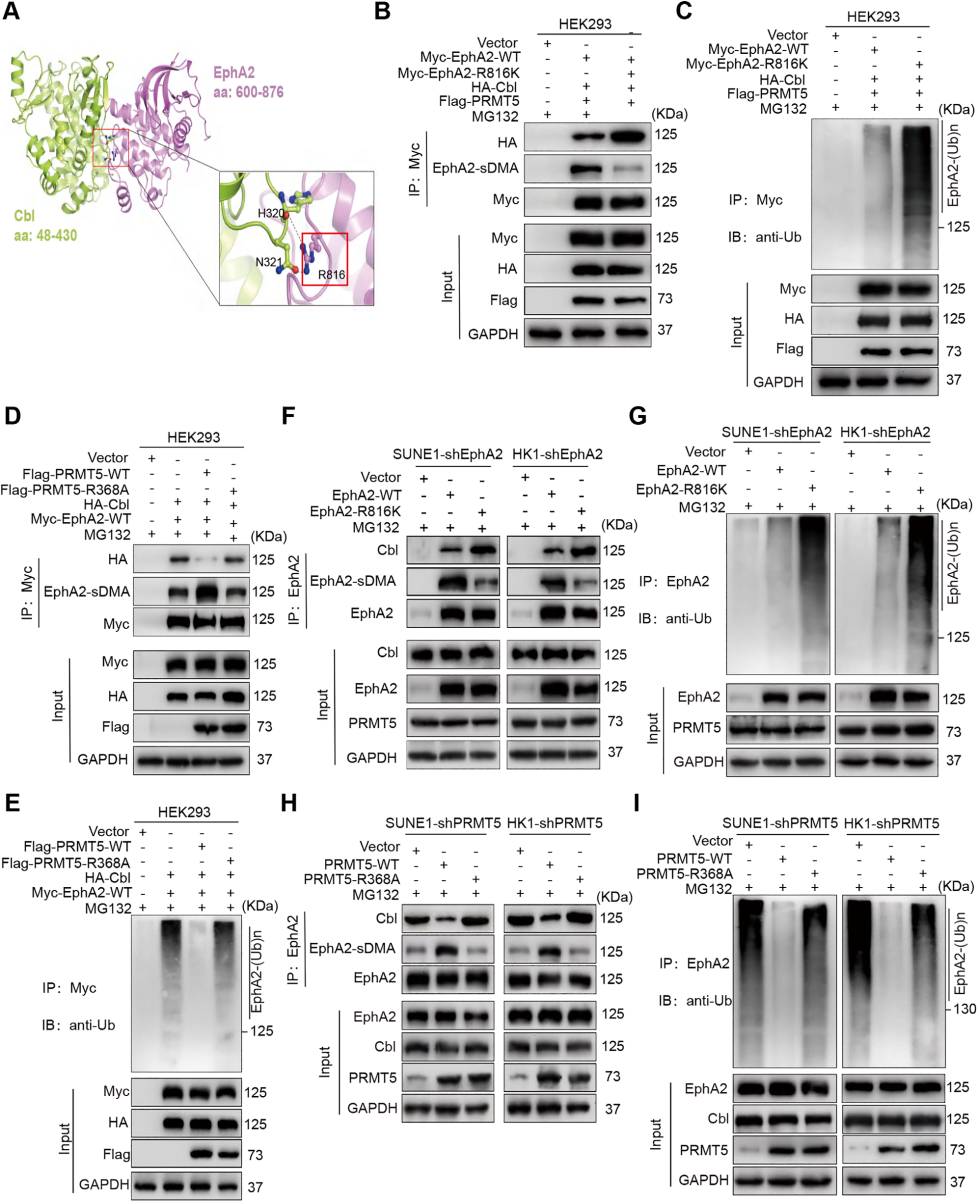
PRMT5‐catalyzed EphA2 R816 dimethylation prevents Cbl‐mediated EphA2 from ubiquitination and degradation. (A) Molecular docking model for Cbl–EphA2 complex. The SH2 domain of Cbl (lime green, Protein Data Bank code: 2Y1M) binds to arginine 816 of EphA2 (pink, Protein Data Bank code: 1MQB). Arginine 816 of EphA2 is shown as a ball‐and‐stick model in the red frame. (B) Co‐IP (co‐immunoprecipitation) showing that WT EphA2, but not methylation inactive mutant EphA2–R816K, increases EphA2 methylation, and decreases Cbl bound to EphA2 in the HEK293 cells transfected with the indicated plasmids. (C) Co‐IP showing that WT EphA2 but not EphA2–R816K decreases EphA2 polyubiquitination in the HEK293 cells transfected with the indicated plasmids. (D) Co‐IP showing that WT PRMT5 but not catalytically inactive mutant PRMT5–R368A increases EphA2 methylation, and decreases Cbl bound to EphA2 in HEK293 cells transfected with the indicated plasmids. (E) Co‐IP showing that WT PRMT5 but not PRMT5–R368A decreases EphA2 polyubiquitination in the HEK293 cells transfected with the indicated plasmids. (F) Co‐IP showing that reintroduction of WT EphA2 but not EphA2–R816K increases EphA2 methylation, and decreases Cbl bound to EphA2 in the endogenous EphA2 knockdown SUNE1 and HK1 NPC cells. (G) Co‐IP showing that reintroduction of WT EphA2 but not EphA2–R816K decreases EphA2 polyubiquitination in the endogenous EphA2 knockdown SUNE1 and HK1 NPC cells. (H) Co‐IP showing that reintroduction of WT PRMT5 but not PRMT5–R368A increases EphA2 methylation, and decreases Cbl bound to EphA2 in the endogenous PRMT5 knockdown SUNE1 and HK1 NPC cells. (I) Co‐IP showing that reintroduction of WT PRMT5 but not PRMT5–R368A decreases EphA2 polyubiquitination in the endogenous PRMT5 knockdown SUNE1 and HK1 NPC cells. shEphA2, EphA2 knockdown by shRNA; shPRMT5, PRMT5 knockdown by shRNA; shCtrl, scramble nontarget shRNA; sDMA, symmetric dimethylarginine; R816, arginine 816; Ub, ubiquitin; WT, wild‐type; IP, immunoprecipitation; IB, immunoblotting.

How does R816 dimethylation increase EphA2 stability? Cbl is a known E3 ubiquitin ligase of EphA2, and its SH2 domain binds to the consensus docking sequence (Y^813^G^814^F^815^R^816^P^817^) of EphA2 kinase domain [35, 36]. Importantly, our docking model for the Cbl–EphA2 complex showed that the SH2 domain of Cbl binds to R816 of EphA2 (Figure 2A). It has been reported that one of the main consequences of arginine methylation is alteration of its binding interactions [4]. Moreover, due to arginine's five potential hydrogen‐bond donors, adding a methyl group will influence the interactions with the hydrogen‐bond acceptors of its interaction partners through steric effects [37]. Therefore, we reasonably infer that R816 methylation could disturb the hydrogen bond and ionic bond formation of Cbl and EphA2, which inhibits the binding of Cbl with EphA2 and its ubiquitination, and then increases EphA2 stability. To verify this, we co‐transfected WT EphA2 or EphA2–R816K and Cbl into HEK293 cells following co‐IP analysis, and observed that the R816K mutation obviously decreased EphA2 methylation and increased binding of Cbl with EphA2 and its ubiquitination (Figure 2B,C). Consistently, transfection of WT PRMT5 but not PRMT5–R368A increased EphA2 methylation and reduced binding of Cbl with EphA2 and its ubiquitination in HEK293 cells (Figure 2D,E). In addition, we generated NPC cell lines with stable endogenous EphA2 knockdown, and observed that reintroduction of WT EphA2 but not EphA2–R816K increased EphA2 methylation, and reduced binding of Cbl with EphA2 and its ubiquitination (Figure 2F,G). Consistently, reintroduction of WT PRMT5 but not PRMT5–R368A increased EphA2 methylation, and decreased binding of Cbl with EphA2 and its ubiquitination in the NPC cells with stable endogenous PRMT5 knockdown (Figure 2H,I). Taken together, our results indicate that PRMT5‐catalyzed EphA2 R816 dimethylation prevents Cbl‐mediated ubiquitination and degradation of EphA2 in the NPC cells.

### 2.3 PRMT5 Promotes NPC Stem Cell Properties by Methylating and Stabilizing EphA2

**FIGURE 3:**
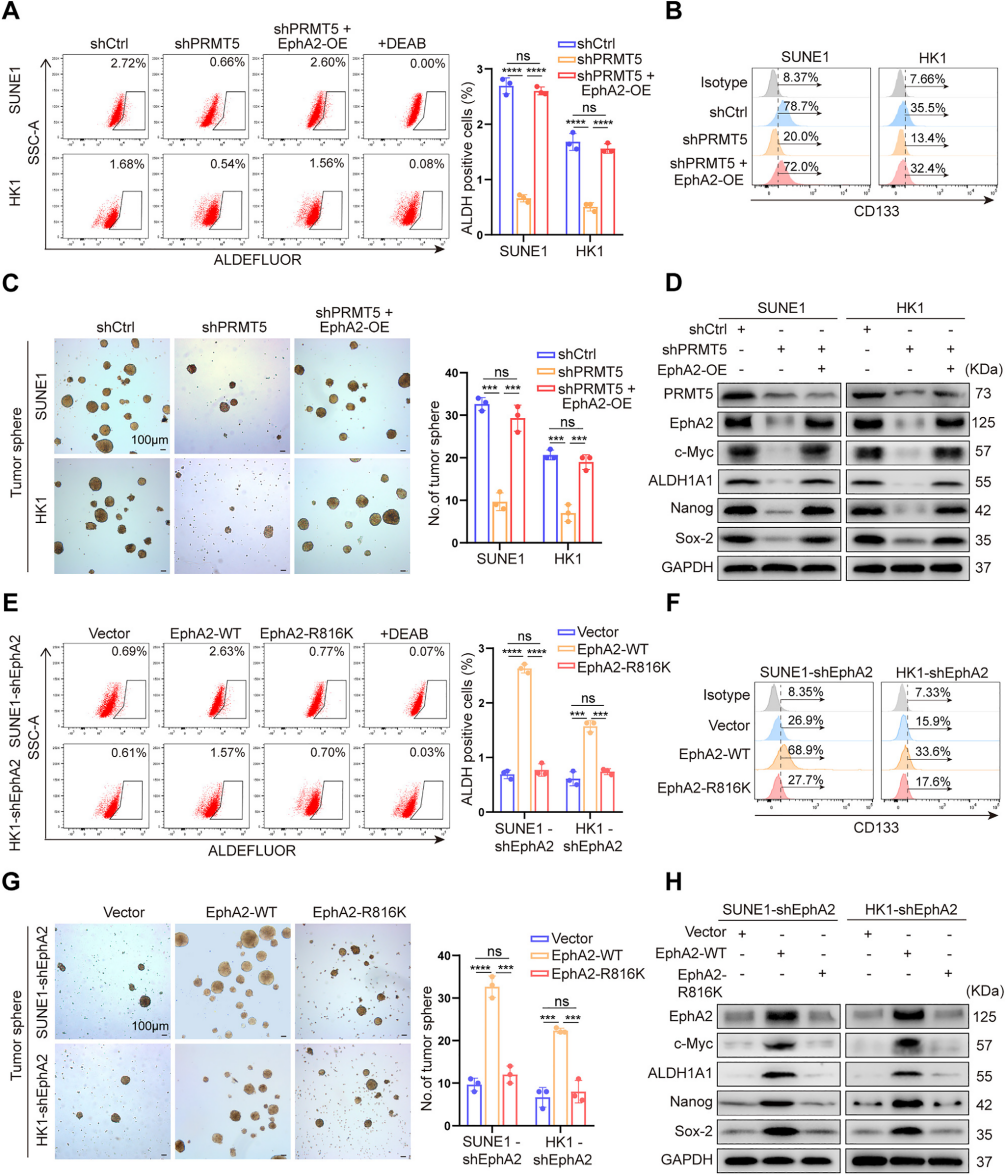
PRMT5 promotes in vitro NPC stem cell properties by methylating and stabilizing EphA2. (A–D) PRMT5 knockdown, PRMT5 knockdown and EphA2 overexpression, and shCtrl control NPC cells are subjected to fluorescence‐activated cell sorting (FACS) for ALDH‐positive cells (A) and CD133‐positive cells (B), tumorsphere formation assay (C), and Western blot analysis for expression levels of c‐Myc, ALDH1A1, Nanog, and Sox‐2 (D). (E–H) Endogenous EphA2 knockdown NPC cells with stable expression of exogenous WT EphA2 or EphA2–R816K, and vector control NPC cells are subjected to fluorescence‐activated cell sorting (FACS) for ALDH‐positive cells (E) and CD133‐positive cells (F), tumorsphere formation assay (G), and Western blot analysis for expression levels of c‐Myc, ALDH1A1, Nanog, and Sox‐2 (H). Scale bars: 100 µm. shEphA2, endogenous EphA2 knockdown by shRNA; EphA2‐OE, EphA2 overexpression; shCtrl, scramble nontarget shRNA; WT, wild‐type. Numbers represent mean ± SD. ***p < 0.001; ****p < 0.0001; ns, no significance.

To test whether PRMT5 increases NPC stem cell properties through methylating and stabilizing EphA2, we generated NPC cell lines with PRMT5 knockdown, or with both PRMT5 knockdown and EphA2 overexpression (Figure S4A), and analyzed whether PRMT5 promotes in vitro NPC stem cell properties by stabilizing EphA2. The results showed that PRMT5 knockdown significantly decreased the percentage of aldehyde dehydrogenase (ALDH)‐positive cells and CD133‐positive cells, tumorsphere formation ability, and the expression levels of four known NPC stem markers (c‐Myc, ALDH1A1, Nanog, and Sox‐2) (Figure 3A–D), and EphA2 overexpression could rescue these cancer stem cell properties in the NPC cell lines with PRMT5 knockdown (Figure 3A–D). Moreover, we generated NPC cell lines stably expressing WT EphA2 or EphA2–R816K using NPC cells with endogenous EphA2 knockdown (Figure S4B), and analyzed the effect of WT EphA2 and EphA2–R816K on in vitro NPC stem cell properties. The results showed that reintroduction of WT EphA2 but not EphA2–R816K could rescue the in vitro NPC stem cell properties (Figure 3E–H). Given the established link between cancer stemness and chemoresistance [32], we further investigated whether the PRMT5–EphA2 axis confers NPC cell chemoresistance. Both methyl thiazolyl tetrazolium (MTT) and plate colony‐formation assay showed that PRMT5 knockdown increased the inhibitory effect of cisplatin on NPC cell proliferation, which was rescued by EphA2 overexpression (Figure S5A,B). In addition, reintroduction of WT EphA2 but not EphA2–R816K decreased the inhibitory effect of cisplatin on the proliferation of endogenous EphA2 knockdown NPC cells (Figure S5C,D). The results indicate that PRMT5‐mediated methylation and stabilization of EphA2 increase NPC cell chemoresistance. Collectively, these data indicate that PRMT5 promotes in vitro NPC stem cell properties via methylating and stabilizing EphA2.

**FIGURE 4:**
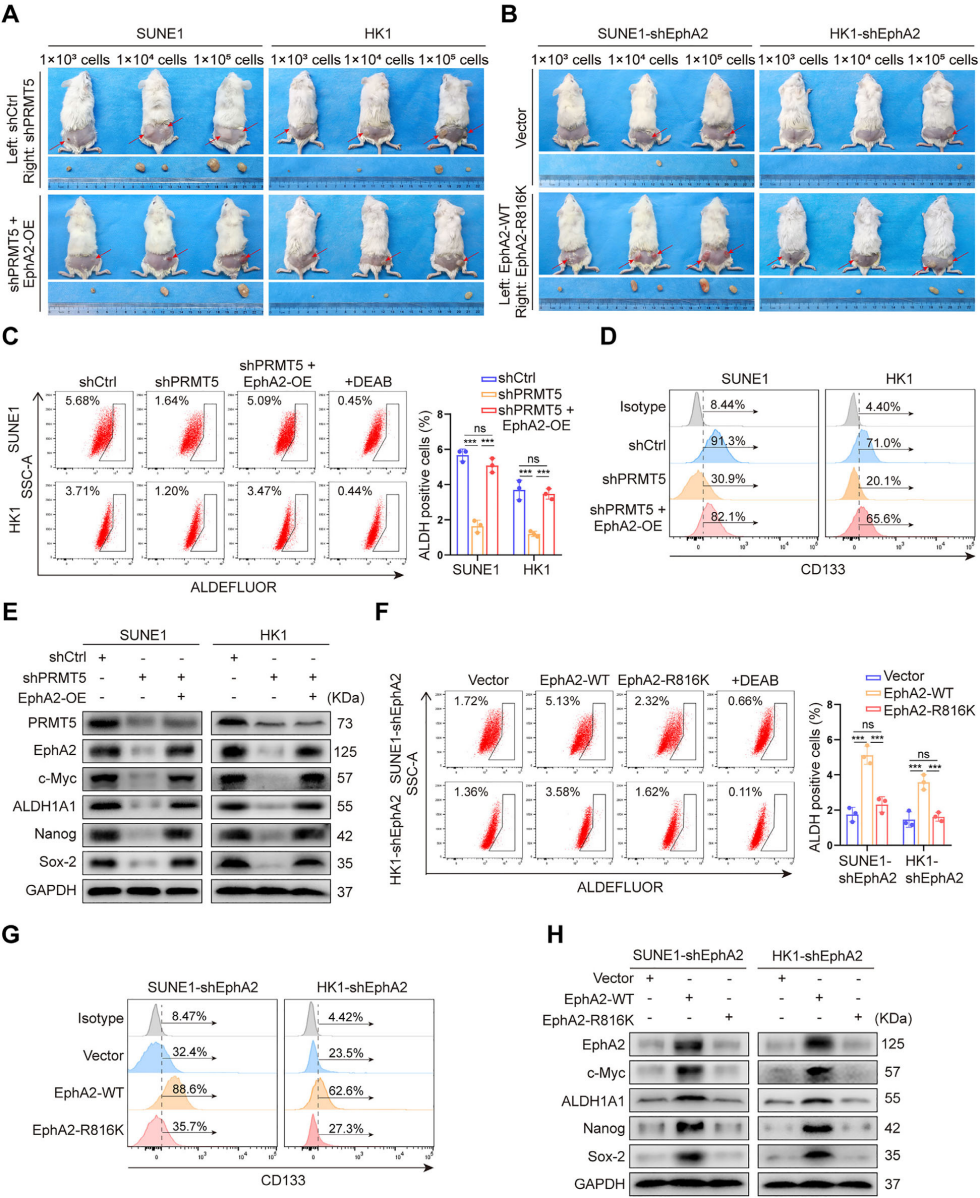
PRMT5 promotes in vivo NPC stem cell properties by methylating and stabilizing EphA2. (A) The representative photographs of xenografts at 5 weeks after subcutaneous implantation of PRMT5 knockdown, PRMT5 knockdown and EphA2 overexpression, and shCtrl control NPC cells, respectively. Eight mice per group. (B) The representative photographs of xenografts at 5 weeks after subcutaneous implantation of endogenous EphA2 knockdown NPC cells expressing exogenous WT EphA2 or EphA2–R816K, and vector control NPC cells, respectively. Eight mice per group. (C–E) Xenografts generated from PRMT5 knockdown, PRMT5 knockdown and EphA2 overexpression, and shCtrl control NPC cells are subjected to FACS for ALDH‐positive cells (C) and CD133‐positive cells (D), and Western blot analysis for expression levels of c‐Myc, ALDH1A1, Nanog, and Sox‐2 (E). (F–H) Xenografts generated from endogenous EphA2 knockdown NPC cells expressing exogenous WT EphA2 or EphA2–R816K and vector control NPC cells are subjected to FACS for ALDH‐positive cells (F) and CD133‐positive cells (G), and Western blot analysis for expression levels of c‐Myc, ALDH1A1, Nanog, and Sox‐2 (H). shEphA2, endogenous EphA2 knockdown by shRNA; EphA2‐OE, EphA2 overexpression; shCtrl, scramble nontarget shRNA; WT, wild‐type. Numbers represent mean ± SD. ***p < 0.001; ns, no significance.

Next, we analyzed whether PRMT5 increases in vivo NPC stem cell properties by methylating and stabilizing EphA2 using tumor‐initiating capacity assay. Serial dilutions of the same NPC cells as the in vitro experiment were subcutaneously implanted into nonobese diabetic severe combined immunodeficiency (NOD‐SCID) mice, and then tumor initiation and growth were monitored. The results showed that PRMT5 knockdown obviously reduced the tumor incidence and growth rate of inoculated NPC cells, and EphA2 overexpression could recover the tumor incidence and growth rate of inoculated PRMT5 knockdown NPC cells in mice (Figure 4A, Figure S6, and Table S1). Consistently, reintroduction of WT EphA2 but not EphA2–R816K could recover the tumor incidence and growth rate of inoculated NPC cells with endogenous EphA2 knockdown in mice (Figure 4B, Figure S6, and Table S2). Moreover, we observed that PRMT5 knockdown significantly decreased the percentage of ALDH‐positive cells and CD133‐positive cells, and the expression levels of the four known NPC stem markers in the xenografts, and EphA2 overexpression could rescue these NPC stem cell properties in the PRMT5 knockdown xenografts (Figure 4C–E). We also observed that reintroduction of WT EphA2 but not EphA2–R816K could rescue these NPC stem cell properties in the endogenous EphA2 knockdown xenografts (Figure 4F–H). Immunohistochemistry (IHC) showed that expression levels of EphA2 and the four known NPC stem markers in the PRMT5 knockdown and EphA2–R816K xenografts were significantly lower relative to their respective control xenografts (Figure S7). Collectively, these data indicate that PRMT5 increases in vivo NPC stem cell properties via methylating and stabilizing EphA2.

### 2.4 PRMT5‐Derived Peptide P20 Degrades EphA2 by Inhibiting the Interaction of PRMT5 and EphA2

Given that PRMT5 binds and stabilizes EphA2, a potential strategy for degrading EphA2 is to disrupt the PRMT5–EphA2 interaction. Protein‐derived peptides tend to bind with target proteins and have the advantage of inhibiting protein–protein interaction [27, 38]. To develop a protein‐derived peptide disturbing PRMT5–EphA2 interaction, we constructed a series of deletion mutants for both proteins to map their interacting regions (Figure S8A), co‐transfected each PRMT5 mutant with WT EphA2, or each EphA2 mutant with WT PRMT5 into HEK293 cells followed by Co‐IP analysis, and observed that both PRMT5 D1–324 and D251–324 mutants failed to bind EphA2 (Figure S8B,C), indicating that PRMT5 251–324 amino acid (aa) residues are responsible for binding EphA2, and both EphA2 D606–906 and D707–807 mutants failed to bind PRMT5 (Figure S8D,E), indicating that EphA2 707–807 aa residues are responsible for binding PRMT5.

Based on the interacting region of PRMT5 and EphA2, the PRMT5–EphA2 complex model was constructed using the HDOCK protein–protein docking. As a result, 10 potential PRMT5–EphA2 docking complexes were yielded, the seventh complex of which exhibited the highest number of residues from the interaction sequences at the protein–protein interface (Figure S9). Specifically, the continuous sequence YLQYLEYLSQNRPPPNAY (280–297aa) of PRMT5 and the continuous sequence KYLANMNYVHR (728–738aa) of EphA2 were found to be involved in interactions, forming multiple interaction hotspots in the seventh complex. Therefore, the seventh complex was determined to represent the most likely PRMT5–EphA2 complex model. Based on the seventh complex, we designed a short peptide (SYLQYLEYLSQNRPPPNAYE) derived from the PRMT5 sequence (279–298aa) to disrupt PRMT5–EphA2 interaction, termed P20 (**P** RMT5‐derived **20** aa‐long peptide). Theoretically, P20 peptide binds to EphA2 and occupies the PRMT5‐binding site on EphA2, then blocks PRMT5 binding and methylating EphA2, resulting in Cbl‐mediated EphA2 degradation.

**FIGURE 5:**
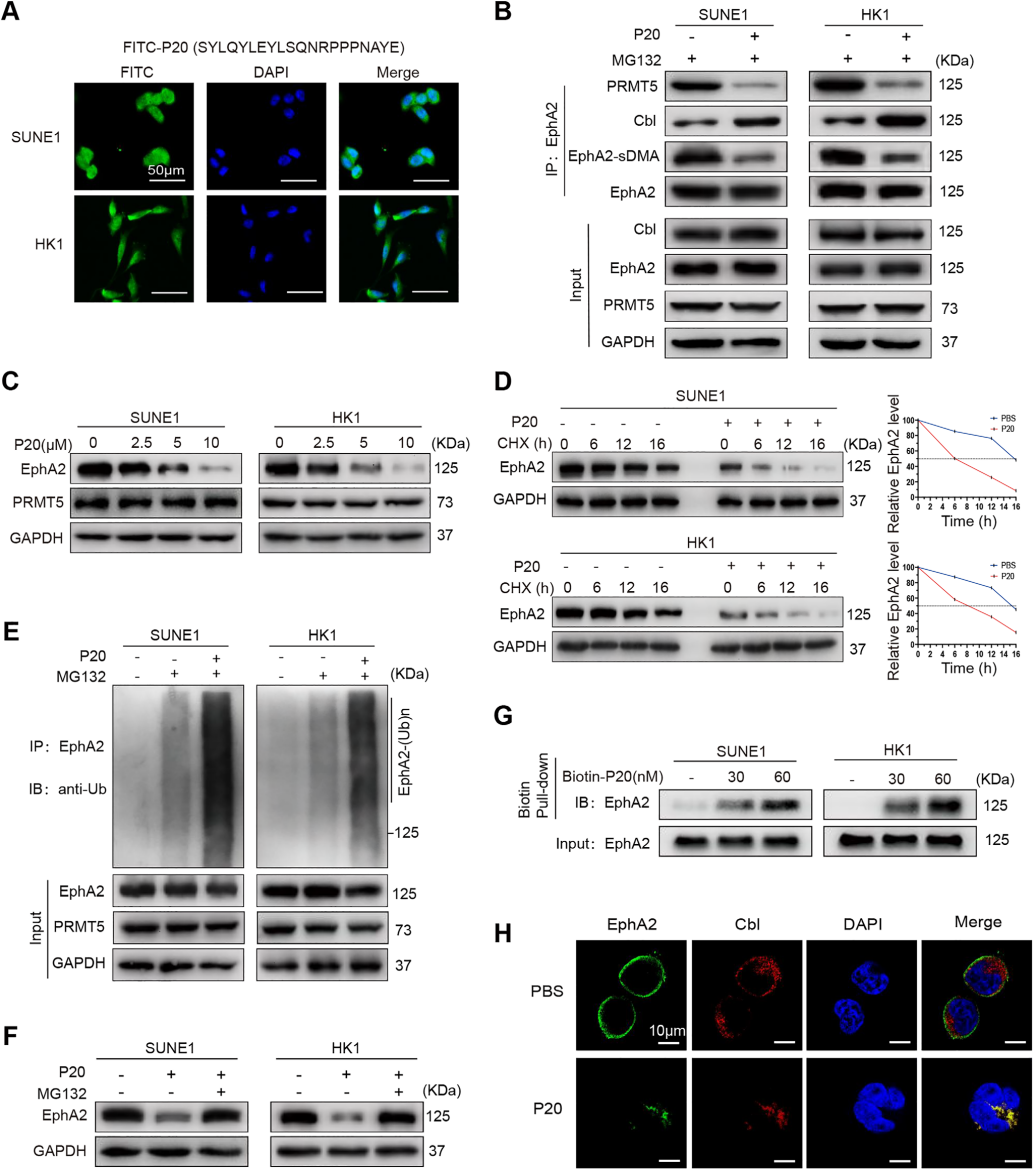
PRMT5‐derived peptide P20 degrades EphA2 by inhibiting the interaction of PRMT5 and EphA2. (A) Efficient cellular uptake of P20 peptide. NPC cells are incubated with 10 µM fluorescein isothiocyanate (FITC)‐labeled P20 for 6 h and observed by fluorescent microscopy. Cell nuclei are stained with 4,6‐diamidino‐2‐phenylindole (DAPI). Scale bars: 50 µm. (B) Co‐IP showing that P20 decreases PRMT5 bound to EphA2 and its methylation, and increases Cbl bound to EphA2 in the NPC cells. (C) Western blot showing that P20 decreases EphA2 levels in the NPC cells in a dose‐dependent manner. (D) Western blot showing that P20 decreases EphA2 protein stability in the NPC cells treated with 20 µg/mL cycloheximide (CHX) for the indicated times. (E) Co‐IP showing P20 increases EphA2 polyubiquitination in the NPC cells. (F) Western blot showing restoration of EphA2 protein levels by proteasome inhibitor MG132 in the NPC cells treated with 10 µM P20. (G) Biotin pull‐down showing P20 bound to EphA2 in the NPC cells. (H) Immunofluorescent staining showing that P20 increases EphA2 (green) internalization and its co‐localization with Cbl (red) in the NPC cells. Scale bars: 10 µm. sDMA, symmetric dimethylarginine.

To test this, the P20 peptide was synthesized. Efficient NPC cellular uptake of P20 was validated by immunofluorescent labeling with fluorescein isothiocyanate (FITC) (Figure 5A). As expected, P20 blocked PRMT5 bound to EphA2, decreased EphA2 methylation, and increased Cbl bound to EphA2 (Figure 5B); P20 reduced EphA2 levels in a dose‐dependent manner (Figure 5C) and EphA2 protein stability (Figure 5D), and increased EphA2 ubiquitination (Figure 5E). Moreover, P20‐decreased EphA2 protein levels were specifically restored by MG132 treatment (Figure 5F). Biotin pull‐down analysis revealed that P20 could pull down EphA2 protein from the NPC cells (Figure 5G), validating that P20 is able to bind EphA2. Moreover, P20 markedly promoted EphA2 internalization and co‐localization of Cbl and EphA2 in the NPC cells (Figure 5H), supporting that P20 degrades EphA2. Collectively, these data demonstrate that P20 blocks PRMT5–EphA2 interaction and targets EphA2 for ubiquitination and degradation in NPC cells.

### 2.5 P20 Peptide Inhibits NPC Stem Cell Properties by Degrading EphA2

**FIGURE 6:**
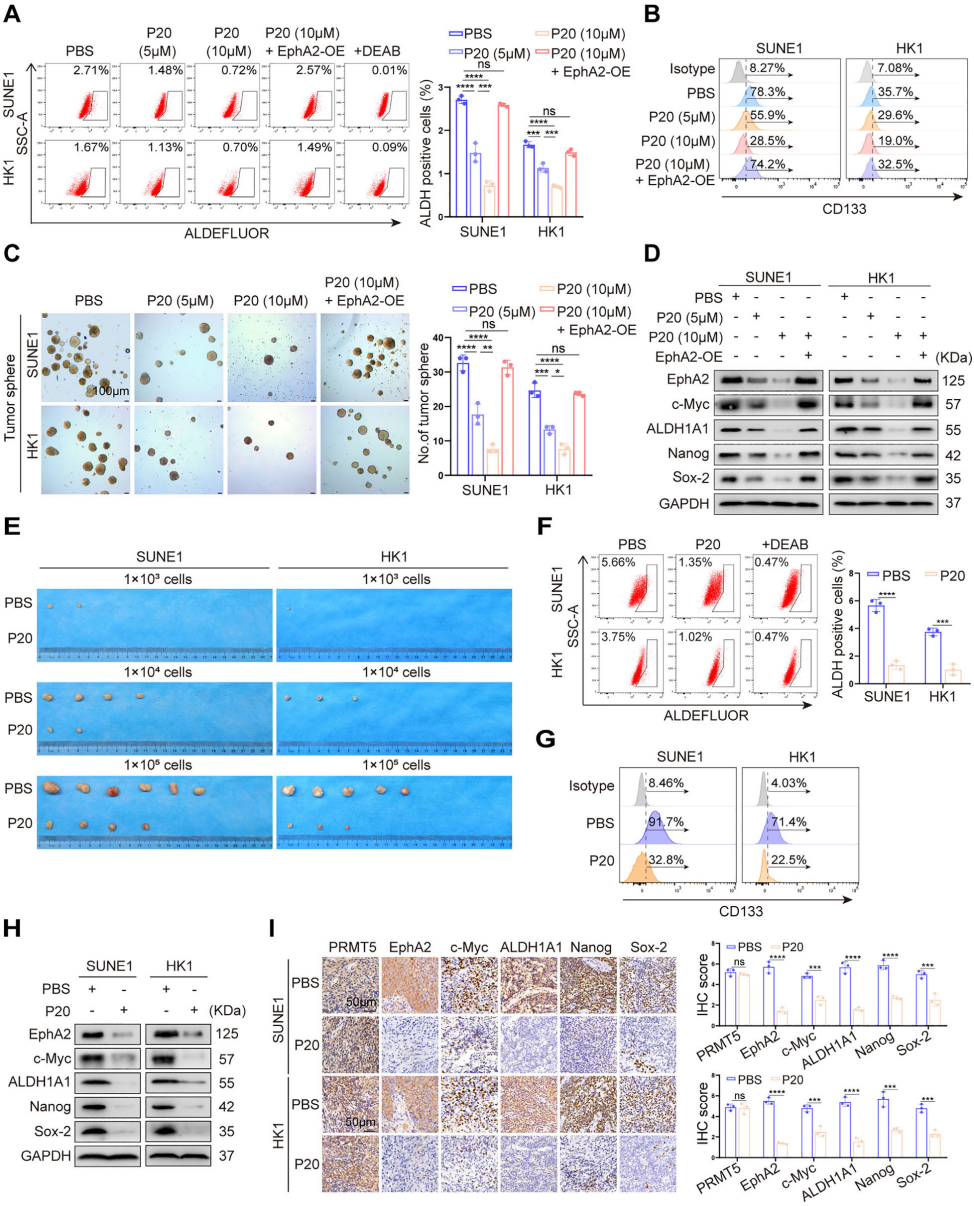
PRMT5‐derived peptide P20 inhibits in vitro and in vivo NPC stem cell properties by degrading EphA2. (A–D) The effect of P20 on in vitro NPC stem cell properties. SUNE1 and HK1 NPC cells and their EphA2 overexpression partners are incubated with P20 for 48 h, and subjected to FACS for ALDH‐positive cells (A) and CD133‐positive cells (B), tumorsphere formation assay (C), and Western blot analysis for expression levels of c‐Myc, ALDH1A1, Nanog, and Sox‐2 (D). (E–I) The effect of P20 on in vivo NPC stem cell properties. The photographs of NPC cell xenografts harvested from each mouse at 5 weeks after subcutaneous implantation, and phosphate‐buffered saline (PBS) treatment serve as the control. Eight mice per group. (E). The xenografts are subjected to FACS for ALDH‐positive cells (F) and CD133‐positive cells (G), Western blot analysis for expression levels of EphA2, c‐Myc, ALDH1A1, Nanog, and Sox‐2 (H), and immunohistochemical staining for expression levels of PRMT5, EphA2, c‐Myc, ALDH1A1, Nanog, and Sox‐2 (I). Scale bars: 50 µm. FACS, fluorescence‐activated cell sorting; DEAB, 4‐diethylaminobenzaldehyde; Isotype, isotype control; EphA2‐OE, EphA2 overexpression. Numbers represent means ± SD. ***p < 0.001; ****p < 0.0001; ns, no significance.

As PRMT5 enhances NPC stemness by binding and stabilizing EphA2, and P20 degrades EphA2 by blocking PRMT5–EphA2 interaction in the NPC cells, we reasonably infer that P20 can inhibit NPC stemness. To test this, NPC cells were treated with P20, followed by analysis of cancer stem cell properties. The results showed that P20 reduced the percentage of ALDH‐positive cells and CD133‐positive cells, tumorsphere formation ability, and the expression levels of the four known NPC stem markers in a dose‐dependent manner, whereas EphA2 overexpression was able to antagonize the effect of P20 on these NPC stem cell properties (Figure 6A–D). Given the association of cancer stemness with chemoresistance [32], we further investigated whether P20 confers NPC cell chemosensitivity. Both MTT and plate colony‐formation assay showed that P20 significantly increased the inhibitory effect of cisplatin on NPC cell proliferation, which was rescued by EphA2 overexpression (Figure S10), indicating that P20 increases NPC cell chemosensitivity via EphA2 mediation. Collectively, these results suggest that P20 inhibits in vitro NPC stem cell properties by degrading EphA2.

To test the impact of P20 on in vivo NPC stemness, serial dilutions of NPC cells were subcutaneously implanted into NOD‐SCID mice. One week post‐inoculation, tumor‐bearing mice were administered P20 (10 mg/kg once daily for consecutive 12 days) via peritoneal injection. After subcutaneous implantation, tumor initiation and growth were monitored. The results showed that P20 significantly decreased the tumor incidence and growth rate of inoculated NPC cells (Figure 6E, Figure S11, and Table S3), indicating that P20 significantly inhibited the tumor‐initiating capacity of inoculated NPC cells in mice. Moreover, we observed that P20 significantly decreased the percentage of ALDH‐positive cells and CD133‐positive cells, and the expression levels of the four known NPC stem markers in the xenografts (Figure 6F–H). IHC also showed that the expression of EphA2 and the four known NPC stem markers in the xenografts treated with P20 was markedly decreased (Figure 6I). Collectively, these data suggest that P20 inhibits in vivo NPC stem cell properties by degrading EphA2.

### 2.6 Correlation of PRMT5 and EphA2 Expression With NPC Patient Prognosis

**FIGURE 7:**
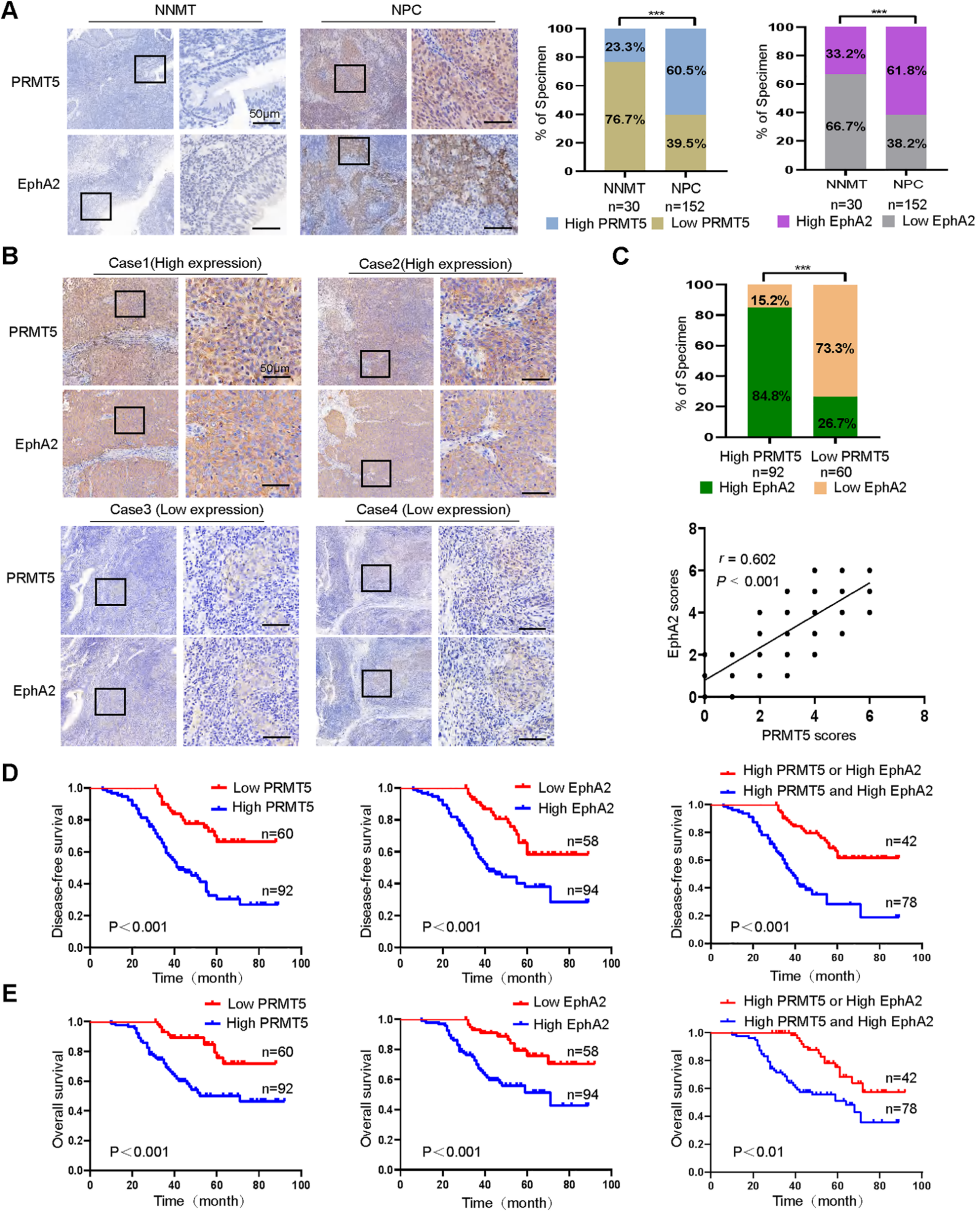
The high expression of both PRMT5 and EphA2 is associated with poor NPC patient prognosis. (A) The expression levels of PRMT5 and EphA2 in the NPC and normal nasopharyngeal mucosal tissues (NNMT). Representative immunohistochemistry (IHC) images of the serial tissue sections are shown on the left, and quantitative data are presented on the right (*p *< 0.001, *χ* ^2^ test). (B and C) A positive correlation between the PRMT5 and EphA2 expression in the NPC tissues. Representative IHC images of high and low expression of PRMT5 and EphA2 in the serial cancer tissue sections are shown in (B), and statistical analysis of the correlation between PRMT5 and EphA2 expressions is presented on the right in (C) (*p* < 0.001, Spearman correlation test). (D and E) Kaplan–Meier survival analysis for 152 patients with NPC according to the levels of PRMT5, EphA2, or both proteins. Log‐rank test is used to calculate the *p*‐value. Scale bars: 50 µm.

To investigate the clinical significance of PRMT5–EphA2 interaction, IHC was performed to examine the expression of both proteins in 152 NPCs and 30 normal nasopharyngeal mucosal tissues (NNMT). The results revealed that the expressions of both proteins in NPC tissues were significantly increased as compared to NNMT (Figure 7A), with a positive correlation in NPC samples (Figure 7B,C). Moreover, higher expression levels of both proteins were associated with lymph node and distant metastasis, and TNM stage and recurrence (Table S4). Survival analysis showed that the patients exhibiting high expression of both proteins had worse disease‐free survival (DFS) and overall survival (OS) as compared to those with high expression of either protein alone (Figure 7D,E). Univariate and multivariate Cox regression analyses identified a combination of PRMT5 and EphA2 as an independent prognostic factor (Table S5). Collectively, these data indicate that PRMT5 might stabilize EphA2 in the NPC tissues, and a combination of PRMT5 and EphA2 could serve as a prognostic marker for NPC patients.

## 3 Discussion

NPC is one of the most common malignancies in southern China and Southeast Asia [31]. NPC stem cells or cancer stem‐like cells are closely associated with tumor recurrence, metastasis, and radiochemotherapy resistance [23, 34, 35]. Therefore, identification of therapeutic targets against NPC stemness and development of novel strategies against the specific targets will be helpful for improving the treatment and prognosis of NPC patients.

PRMT5 is not only associated with the development and progression of various malignancies [5, 6, 7, 8, 9, 10] but also increases cancer stemness [12, 13]. Although some reports have investigated how PRMT5 promotes cancer stem cell properties, the underlying mechanisms still need to be elucidated. In the present study, we found that PRMT5 bound EphA2 and catalyzed dimethylation of EphA2 at R816, which inhibited Cbl‐mediated EphA2 ubiquitination and degradation, thereby promoting NPC stem cell properties. Our study reveals a novel mechanism of PRMT5‐promoting cancer stemness, that is, the interplay between PRMT5‐catalyzed arginine dimethylation and Cbl‐mediated ubiquitination of EphA2 increases NPC stemness.

As PRMT5 overexpression is associated with the development, progression, and stemness of cancers, it becomes a prominent target in anticancer drug development [15]. Numerous pharmaceutical companies have actively engaged in the development of compounds targeting PRMT5, and the PRMT5 inhibitors that inhibit methyltransferase activity have entered clinical trials [39, 40, 41]. PRMT5 also plays an important role in normal cells, including neural and hematopoietic stem cells [11, 42], leading to the higher toxicity and side effects of the PRMT5 inhibitors. Therefore, identifying novel approaches for targeting PRMT5 will provide alternative strategies for cancer treatment.

EphA2 is overexpressed and promotes cancer growth, metastasis, and stemness in various cancers, including NPC [17, 18, 19, 20, 21, 22, 23]. Therefore, downregulating EphA2 has been considered as an anticancer strategy [24, 25]. In this study, our results showed that PRMT5 promoted NPC stem cell properties by binding and stabilizing EphA2, indicating that PRMT5–EphA2 interaction is a therapeutic target for inhibiting NPC stemness. For this purpose, we developed a PRMT5‐derived peptide P20, which bound EphA2 and occupied the PRMT5‐binding site in EphA2, disrupted PRMT5–EphA2 interaction, targeted EphA2 for ubiquitination and degradation, and inhibited in vitro and in vivo NPC stem cell properties. These results indicate that blocking PRMT5–EphA2 interaction by P20 represents a new strategy for NPC treatment. As the anti‐NPC effect of P20 originates from blocking PRMT5–EphA2 interaction, P20 does not inhibit PRMT5’s methyltransferase activity, and also does not interfere with the functions of other PRMT5's targets. Therefore, we think that P20 is a promising approach for treating NPC without toxicity and side effects. Peptide drugs combine the advantages of traditional chemical drugs and protein‐based therapeutics [43], which have broad development prospects. However, peptides suffer from drawbacks such as instability and short half‐life. Therefore, it is essential to evaluate and improve P20's stability for potential upcoming clinical trials.

Our data revealed that PRMT5‐stabilizing EphA2 played a crucial role in maintaining NPC stem cell properties. Consistent with this observation, our IHC results showed that expressions of both PRMT5 and EphA2 were significantly increased in NPC tissues relative to NNMT, with a positive correlation in NPC tissues. NPC patients exhibiting high expression of both proteins had worse DFS and OS compared to those with high expression of either protein alone, indicating that PRMT5 might stabilize EphA2 in the NPC tissues, and a combination of PRMT5 and EphA2 could serve as a prognostic marker for NPC patients.

In this study, there are some limitations. Although the effect and mechanism of PRMT5‐stabilizing EphA2 and P20 peptide‐inhibiting NPC stem cell properties have been thoroughly validated in the preclinical studies, these findings should be further confirmed in more complex in vivo models that recapitulate the human tumor microenvironment. In addition, the pharmacokinetics of P20 necessitate further investigation for its clinical translation.

## 4 Conclusion

In summary, our present study identifies PRMT5 as an arginine methyltransferase of EphA2 and discovers that PRMT5‐catalyzed EphA2 methylation at R816 stabilizes EphA2 via inhibiting Cbl‐mediated EphA2 ubiquitination and degradation, which promotes NPC stemness. We also develop a PRMT5‐derived peptide P20 disturbing PRMT5–EphA2 interaction and targeting EphA2 degradation, which inhibits NPC stemness. Our data reveal the function and mechanism of PRMT5–EphA2 interaction in NPC, and present a potential strategy for treating NPC with a peptide.

## 5 Materials and Methods

### 5.1 Human NPC Specimens

A total of 152 archival NPC tissues fixed in formalin and embedded in paraffin were collected between January 2016 and January 2019 from Xiangya Hospital of Central South University at the time of diagnosis, before any therapy. Thirty normal nasopharyngeal mucosal tissues were also obtained during the same period. The clinicopathologic characteristics of specimens are shown in Table S6. For additional information, refer to the Supporting Information Materials and Methods section.

### 5.2 Animal Experiments

Four‐week‐old nonobese diabetic/severe combined immunodeficient (NOD/SCID) mice were purchased from Hunan Silaike Jingda Laboratory Animal Co. Ltd. and maintained in the specific‐pathogen‐free facility of the Experimental Animal Center at Xiangya Hospital of Central South University. The in vivo tumor‐initiating capacity of indicated NPC cells was examined in mice. For additional information, refer to the Supporting Information Materials and Methods section.

### 5.3 Cell Lines

The NPC SUNE1 and HK1 cell lines and the embryonic kidney HEK293 cell line were obtained from the Shanghai Chinese Academy of Sciences Cell Bank in March 2022. The cell lines were authenticated by short tandem repeat profiling and confirmed to be free of *Mycoplasma* contamination.

### 5.4 Establishment of Stably Transfected NPC Cell Lines With the Expression Changes of PRMT5 and EphA2

NPC cells were transduced with the indicated lentiviral particles following the uniform procedure, or transfected with the indicated plasmids using Lipofectamine 2000, then selected using antibiotics for 2 weeks. All established NPC cell lines were confirmed by Western blot analysis.

### 5.5 Immunoprecipitation Conjugated With Mass Spectrometry Analysis

IP‐MS was performed to identify the methylated arginines of EphA2 protein in the NPC cells as previously described [44]. For additional information, refer to the Supporting Information Materials and Methods section.

### 5.6 Duolink Proximity Ligation Assay (PLA)

The Duolink In Situ Red Starter Kit Mouse/Rabbit kit was used to detect PRMT5 and EphA2 interaction following our previous experimental procedures [30]. For additional information, refer to the Supporting Information Materials and Methods section.

### 5.7 Western Blot

Cells and xenografts lysed in radio immunoprecipitation assay (RIPA) buffer were used to extract proteins. For each sample, 50 µg of proteins was subjected to sodium dodecyl sulfate‐polyacrylamide gel electrophoresis (SDS‐PAGE) separation and subsequently transferred to a polyvinylidene fluoride (PVDF) membrane. Blots were blocked in blocking buffer, and then incubated with primary antibodies for 12 h at 4°C, followed by incubation with horseradish peroxidase (HRP)‐conjugated anti‐rabbit IgG or HRP‐conjugated anti‐mouse IgG for 2 h at room temperature. Enhanced chemiluminescent reagents were used for signal visualization.

### 5.8 Co‐Immunoprecipitation

Co‐immunoprecipitation (Co‐IP) was performed to detect protein–protein interaction and EphA2 ubiquitination. Briefly, proteins were precleared with Protein A/G‐Sepharose 4B, and the clarified supernatants were incubated with the indicated antibodies and Protein A/G‐Sepharose 4B overnight at 4°C. After washing with RIPA buffer five times, beads were boiled in 2× SDS‐PAGE loading buffer for 5 min to elute protein complexes, followed by SDS‐PAGE separation and immunoblotting with specific antibodies.

### 5.9 GST Pull‐Down Assay

The GST pull‐down assay was performed to detect the direct interaction between PRMT5 and EphA2 following our previous experimental procedures [30]. For additional information, refer to the Supporting Information Materials and Methods section.

### 5.10 In Vitro Methylation Analysis

The methylation level of purified EphA2 protein was detected by in vitro methylation analysis as previously described [13]. For additional information, refer to the Supporting Information Materials and Methods section.

### 5.11 Biotin Pull‐Down Assay

The interaction of peptide and EphA2 was detected using biotin pull‐down assay following our previous experimental procedures [26]. For additional information, refer to the Supporting Information Materials and Methods section.

### 5.12 Quantitative Real‐Time Polymerase Chain Reaction (qRT‐PCR)

qRT‐PCR was used to detect the mRNA levels of PRMT5 and EphA2 in the indicated cells following our previous experimental procedures [23]. The primers are shown in Table S7. For additional information, refer to the Supporting Information Materials and Methods section.

### 5.13 Tumorsphere Formation Assay

Tumorsphere formation assay was performed to evaluate in vitro NPC stem cell‐like properties, as previously described by us [23]. Briefly, 1 × 10^3^ NPC cells were inoculated into each well in six‐well plates with an ultralow attachment surface, and cultured in serum‐free DMEM/F12 medium supplemented with 20 ng/mL bFGF, 20 ng/mL EGF, and 20 µL/mL B27 supplement. For P20 peptide treatment, 0–10 µM P20 was added to medium and refreshed every day. After 7–9 days of culture, the number of spheres larger than 100 µm was counted under an inverted optical microscope (Leica DMI4000) and photographed.

### 5.14 Flow Cytometric Analysis

Both the aldehyde dehydrogenase (ALDH)‐positive cell population and the CD133‐positive cell population were analyzed by flow cytometry. For additional information, refer to the Supporting Information Materials and Methods section.

### 5.15 Methyl Thiazolyl Tetrazolium Assay

Methyl thiazolyl tetrazolium (MTT) assay was performed to detect the effect of the PRMT5–EphA2 axis and P20 peptide on NPC cell chemosensitivity, as previously described by us [45]. Briefly, cells were cultured in 96‐well culture plates (1 × 10^4^/well) for 12 h, and treated with cisplatin for 48 h, followed by incubation with MTT solution. The absorbance of each well was read with a Bio‐Tek Instruments EL310 Microplate Autoreader at 570 nm. Three independent experiments were performed.

### 5.16 Plate Colony‐Formation Assay

Plate colony‐formation assay was performed to detect the effect of PRMT5–EphA2 axis and P20 peptide on NPC cell chemosensitivity, as previously described by us [46]. Briefly, cells were cultured in six‐well culture plates (2 × 10^3^/well) for 12 h, and treated with cisplatin for 12 days. Surviving colonies were fixed with 4% paraformaldehyde and stained with 0.5% crystal violet. The number of colonies was counted under a Leica DMI4000 microscope. The assay was performed three times.

### 5.17 Immunofluorescent Staining

The subcellular location of EphA2 and Cbl proteins in the indicated cells was detected using immunofluorescent staining following our previous experimental procedures [26]. For additional information, refer to the Supporting Information Materials and Methods section.

### 5.18 Immunohistochemistry

The expression levels of PRMT5, EphA2, c‐Myc, ALDH1A1, Nanog, and Sox2 in the formalin‐fixed and paraffin‐embedded tissues were detected using immunohistochemistry following our previous experimental procedures [26]. For additional information, refer to the Supporting Information Materials and Methods section.

### 5.19 Molecular Docking

Molecular docking was performed to identify the amino acid residues of Cbl and EphA2 interaction [47], and to screen the protein‐derived peptide disturbing PRMT5–EphA2 interaction [48]. For additional information, refer to the Supporting Information Materials and Methods section.

### 5.20 Statistical Analysis

All analyses were performed using IBM SPSS Statistics 22.0, and data visualization was generated by GraphPad Prism 8.0. The data are represented as mean ± SD. Differences between the two groups were evaluated using either Student's *t*‐test or the Mann–Whitney *U* test, as appropriate. For multiple group comparisons, one‐way ANOVA followed by Tukey's post hoc test was applied. Classification variables were compared by the chi‐square test. Correlation analyses were performed using Spearman's rank correlation, with both correlation coefficients and statistical significance reported. Survival outcomes were analyzed using the Kaplan–Meier method, and statistical differences were determined by the log‐rank test. *p*‐value less than 0.05 was considered statistically significant.

## Author Contributions

Zheng‐Zheng Yu and Xue‐Li Mao designed this project, conducted experiments, and acquired data. Shan‐Shan Lu, Ruo‐Huang Lu, Wei Zhu, Di Wu, and Hong Yi conducted experiments and acquired data. Wei Huang, Qi Wen, and Guo‐Xiang Lin analyzed data and provided reagents. Ting Zeng, Yun‐Xi Peng, Li Yuan, Ting Ran, and Juan Feng analyzed and interpreted data. Jinwu Peng and Zhi‐Qiang Xiao designed and supervised this project, and wrote the manuscript. All authors have read and approved the final version of the manuscript.

## Funding

This work was supported by grants from the National Natural Science Foundation of China (Grant Numbers: 82573319, 82272798, 82302976).

## Ethics Statement

This study was approved by the Ethics Committee of Xiangya Hospital, Central South University (approval number: 2022020312). Animal experiments were performed according to the Guide for the Care and Use of Laboratory Animals of Xiangya Hospital, Central South University, with the approval of the Institutional Animal Ethics Committee (approval number: 2022020312). Given that only archived cancer tissues were used in this study, the ethics committee waived the need for consent, and patient records/information were analyzed anonymously.

## Conflicts of Interest

The authors declare no conflicts of interest.

## Supporting information




**Supporting Table 1**: Tumor‐initiation capacity of NPC cell lines with PRMT5 knockdown or PRMT5 knockdown and EphA2 overexpression.
**Supporting Table 2**: Tumor‐initiation capacity of NPC cell lines with stable expression of exogenous WT EphA2 or EphA2–R816K.
**Supporting Table 3**: Tumor‐initiation capacity of the NPC cell lines in tumor‐bearing mice that received P20 treatment.
**Supporting Table 4**: Correlations between the expression of the two proteins and clinicopathological characteristics in NPC (*n* = 152).
**Supporting Table 5**: Univariate and Cox multivariate analyses of prognostic factors for disease‐free survival (*n* = 152).
**Supporting Table 6**: The clinicopathological characteristics of 152 patients with NPC.
**Supporting Table 7**: The primers used for the amplification of the genes by qRT‐PCR.
**Supporting Figure 1**: PRMT5 binds and stabilizes EphA2 protein by inhibiting its ubiquitination and degradation.
**Supporting Figure 2**: PRMT5 inhibitor PJ‐68 decreases EphA2 stability by the ubiquitin proteasome pathway in NPC cells.
**Supporting Figure 3**: PRMT5 increases EphA2 protein stability by catalyzing dimethylation of EphA2 at R816.
**Supporting Figure 4**: Establishment of stably transfected NPC cell lines.
**Supporting Figure 5**: PRMT5 promotes NPC cell chemoresistance by methylating and stabilizing EphA2.
**Supporting Figure 6**: Tumor‐initiating capacity assay showing the effect of PRMT5‐methylating and ‐stabilizing EphA2 on in vivo NPC cell stemness.
**Supporting Figure 7**: Immunohistochemistry (IHC) showing the effect of PRMT5 knockdown (A) and methylation inactivation mutant EphA2–R816K (B) on the expression of EphA2, c‐Myc, ALDH1A1, Nanog, and Sox‐2 in the xenografts. Representative IHC images are shown on the top, and statistical analysis is presented on the bottom. Scale bar: 50 µm. shEphA2, EphA2 knockdown by shRNA; shCtrl, scramble nontarget shRNA; EphA2‐OE, EphA2 overexpression; WT, wild‐type. Numbers represent mean ± SD. ***p < 0.001; ****p < 0.0001; ns, no significance.
**Supporting Figure 8**: Mapping of the binding regions of PRMT5 and EphA2.
**Supporting Figure 9**: Molecular docking model for PRMT5–EphA2 complex.
**Supporting Figure 10**: P20 peptide increases NPC cell chemosensitivity.
**Supporting Figure 11**: Tumor‐initiating capacity assay showing the effect of P20 peptide on in vivo NPC cell stemness.

## Data Availability

The mass spectrometry and proteomics data have been deposited to the ProteomeXchange Consortium (through the PRIDE partner repository) with the dataset identifier PXD015242.
