# Supplementary material for: PRMT5 Methylates and Stabilizes EphA2 via Inhibiting Its Ubiquitination and Degradation to Promote Nasopharyngeal Carcinoma Stem Cell Properties
Source: MedComm (2020). 2026 Mar 28;7(4):e70697. doi: 10.1002/mco2.70697 (PMC13042777; doi:10.1002/mco2.70697)
Supplement: Supplementary file 1 — Supporting Table 1: Tumor‐initiation capacity of NPC cell lines with PRMT5 knockdown or PRMT5 knockdown and EphA2 overexpression. Supporting Table 2: Tumor‐initiation capacity of NPC cell lines with stable expression of exogenous WT EphA2 or EphA2–R816K. Supporting Table 3: Tumor‐initiation capacity of the NPC cell lines in tumor‐bearing mice that received P20 treatment. Supporting Table 4: Correlations between the expression of the two proteins and clinicopathological characteristics in NPC (n = 152). Supporting Table 5: Univariate and Cox multivariate analyses of prognostic factors for disease‐free survival (n = 152). Supporting Table 6: The clinicopathological characteristics of 152 patients with NPC. Supporting Table 7: The primers used for the amplification of the genes by qRT‐PCR. Supporting Figure 1: PRMT5 binds and stabilizes EphA2 protein by inhibiting its ubiquitination and degradation. Supporting Figure 2: PRMT5 inhibitor PJ‐68 decreases EphA2 stability by the ubiquitin proteasome pathway in NPC cells. Supporting Figure 3: PRMT5 increases EphA2 protein stability by catalyzing dimethylation of EphA2 at R816. Supporting Figure 4: Establishment of stably transfected NPC cell lines. Supporting Figure 5: PRMT5 promotes NPC cell chemoresistance by methylating and stabilizing EphA2. Supporting Figure 6: Tumor‐initiating capacity assay showing the effect of PRMT5‐methylating and ‐stabilizing EphA2 on in vivo NPC cell stemness. Supporting Figure 7: Immunohistochemistry (IHC) showing the effect of PRMT5 knockdown (A) and methylation inactivation mutant EphA2–R816K (B) on the expression of EphA2, c‐Myc, ALDH1A1, Nanog, and Sox‐2 in the xenografts. Representative IHC images are shown on the top, and statistical analysis is presented on the bottom. Scale bar: 50 µm. shEphA2, EphA2 knockdown by shRNA; shCtrl, scramble nontarget shRNA; EphA2‐OE, EphA2 overexpression; WT, wild‐type. Numbers represent mean ± SD. ***p < 0.001; ****p < 0.0001; ns, no significance. Supportin [file MCO2-7-e70697-s001.pdf]

PRMT5 methylates and stabilizes EphA2 via inhibiting its ubiquitination and degradation to promote nasopharyngeal carcinoma stem cell properties

Zheng-Zheng Yu[1, 2, 3, 4#], Xue-Li Mao[1, 2, 3, 4#], Shan-Shan Lu[2, 3, 4], Ruo-Huang Lu[5], Wei Zhu[1, 4], Di Wu[2, 3, 4], Hong Yi[2, 3, 4], Wei Huang[2, 3, 4], Qi Wen[2, 3, 4], Guo-Xiang Lin[2, 3, 4], Ting Zeng[2, 3, 4], Yun-Xi Peng[2, 3, 4], Li Yuan[6], Ting Ran[7], Juan Feng[2, 3], Jinwu Peng[1, 4*], Zhi-Qiang Xiao[1, 2, 3, 4*]

[1]Department of Pathology, Xiangya Hospital, Central South University, Changsha 410008, China

[2]Research Center of Carcinogenesis and Targeted Therapy, Xiangya Hospital, Central South University, Changsha 410008, China

[3]The Higher Educational Key Laboratory for Cancer Proteomics and Translational Medicine of Hunan Province, Xiangya Hospital, Central South University, Changsha 410008, China

[4]National Clinical Research Center of Geriatric Disorders (Xiangya Hospital), Central South University, Changsha 410011, China

[5]Department of Oral Medicine, The Third Xiangya Hospital, Central South University, Changsha 410013, China

[6]Department of Nuclear Medicine, The Third Xiangya Hospital, Central South University, Changsha 410013, China

[7]Bioland Laboratory (Guangzhou Regenerative Medicine and Health GuangDong Laboratory), Guangzhou 510530, China

Running Head: PRMT5-EphA2 interaction and cancer stemness

[#]These authors contributed equally

*Correspondence: Zhi-Qiang Xiao (zhiqiangxiao@csu.edu.cn), Research Center of Carcinogenesis and Targeted Therapy, Xiangya Hospital, Central South University, Changsha 410008, China; and Jinwu Peng (jinwupeng@csu.edu.cn), Department of Pathology, Xiangya Hospital, Central South University, Changsha 410008, China.

Supplementary Information

Supplementary Materials and Methods.

Supplementary Table S1-7

Supplementary Figure S1-11

Supplementary Materials and Methods

Human NPC specimens

A total of 152 archival NPC tissues fixed in formalin and embedded in paraffin were collected between Jan 2016 and Jan 2019 from Xiangya Hospital of Central South University at the time of diagnosis before any therapy. According to the 2022 WHO classification, all NPCs were pathologically diagnosed as non-keratinizing carcinomas. The TNM stage of the tumors was classified and reclassified in basis of the 2017 AJCC staging system. All the patients received radiotherapy and chemotherapy following a uniform guideline, and information on clinicopathological features and prognoses of the patients were collected and analyzed retrospectively. The follow-up period ranged from 10 to 92 months, with a mean duration of 38.72 ± 13.39 months. Overall survival was defined as the time from the start of treatment to the date of cancer-related death or the date of last follow-up for those who remained alive. Disease-free survival was defined as the time from the end of treatment to the date of pathological diagnosis or clinical evidence of local recurrence and/or distant metastasis. The clinicopathologic features of the patients are presented in Tables S6.

Animal experiments

To test whether PRMT5 promotes *in vivo* NPC cell stem properties by methylating and stabilizing EphA2, serial dilutions (1×10^3, 1×10^4 and 1×10^5) of NPC cells with stable PRMT5 knockdown, stable PRMT5 knockdown and EphA2 overexpression, and stable expression of exogenous EphA2 or EphA2-R816K were subcutaneously injected into NOD-SCID mice respectively (n=8 mice each).

To evaluate the effect of P20 peptide on *in vivo* NPC cell stem properties, serial dilutions (1×10^3, 1×10^4 and 1×10^5) of the NPC cells were subcutaneously injected into NOD-SCID mice. 7 days after the inoculation, mice were divided randomly into

two groups (n=8 mice each), and P20 peptide (10 mg/kg once daily for continuous 12 days) was intraperitoneally injected to mice, and PBS was intraperitoneally injected into mice as a control.

The mice were observed daily for the development of detectable tumors. Tumor sizes were measured using an electronic caliper each day, and their volumes were calculated using the formula ($length \times width^2/2$). The mice were sacrificed at 35 days post-inoculation, and their tumor were collected and weighted using double-blinded evaluation. To perform flow cytometry analysis, single-cell suspensions of the tumors were prepared. Meanwhile, partial tumor samples were rapidly frozen in liquid nitrogen for Western blot analysis and immunohistochemistry.

Plasmids

pBabepuro-EphA2 plasmid, and GV101 expressing EphA2 shRNA have been described previously by us [1], and the shRNA target sequence locating in the EphA2 mRNA 3’UTR is 5’-CAGCCTTCGGACAGACATA-3’. GV505 expressing full length EphA2 or R816K mutant EphA2, and wild-type and deletion mutant PRMT5 with Flag tag, and the full-length and deletion mutant EphA2 with Myc tag were constructed by Genechem (Shanghai, China), and have been verified by DNA sequencing. Plasmid expressing HA-Ub, HA-UbK48, HA-UbK63 and PLKO.1 expressing PRMT5 shRNA were kindly provided by Prof. Ceshi Chen (Kunming institute of zoology, Chinese academy of science, China). HA-Cbl expression plasmid was the gift from Prof. Song-Shu Meng (Dalian Medical University, China).

Antibodies and reagents

Antibodies against EphA2 (6997), PRMT5 (79998), sDMA (13222), HA (3724), Cbl (2747), ALDH1A1 (54135) and Ubiquitin (43124), and HRP-conjugated goat anti-rabbit IgG (7074), and HRP-conjugated goat anti-mouse IgG (7076) were

purchased from Cell Signaling Technology. Anti-EphA2 antibody (sc-398832) and Anti-Ubiquitin antibody (P4D1) (sc-8017) were purchased from Santa Cruz Biotechnology. Antibodies against GAPDH (A19056), GST-Tag (AE077) and Myc-Tag (AE070), c-Myc (A19032), Sox-2 (A0561) and Nanog (A3232) were purchased from Abclonal Technology. Anti-Flag-tag antibody (F1804), SAM (A4377), collagenase VI (C5138), hyaluronidase (H3506), DNase I (DN25) and Duolink® *In Situ* PLA kit (DUO92101) were purchased from Sigma-Aldrich. APC mouse anti-human CD133 (566596), IgG1κ Isotype (554681) and Human BD Fc Block™ (564220) were purchased from BD Biosciences. PerCP/Cyanine5.5 anti-mouse H-2K^d antibody (116617) and Zombie Aqua™ Fixable Viability kit (423101) were purchased from BioLegend. 4',6-diamidino-2'-phenylindole (DAPI) (G1012) was purchased from Servicebio. Reverse transcription kit (A5003) was purchased from Promega. QuantiFast SYBR green PCR kit (204057) was purchased from Qiagen. MG-132 (HY-13259) and Cycloheximide (HY-12320) were purchased from MedChemExpress. Protein G/A-Sepharose™ 4B (82085), Streptavidin agarose (20357), Trizol (15596026) and Lipofectamine 2000 (11668019), bFGF(PHG0266), B27 supplement (17504044), EGF (RP8661) and DMEM/F-12 (11320033) were purchased from ThermoFischer Scientific. ALDEFLUOR™ kit (01700) was purchased from STEMCELL Technologies. DyLight® 488 anti-mouse IgG (DI-2788) and DyLight® 594 anti-Rabbit IgG (DI-1794) were purchased from Vector Laboratories.

PRMT5-dereived peptide

PRMT5-derived 20-mer peptide (279-298aa) (SYLQYLEYLSQNRPPPNAYE), FITC-labeled PRMT5-derived 20-mer peptide, and biotin-labeled PRMT5-derived 20-mer peptide were synthesized by ChinaPeptides (Suzhou, China).

Immunoprecipitation conjugated with mass spectrometry analysis (IP-MS)

IP-MS was preformed to identify the methylated arginines of EphA2 protein in the NPC cells as previously described [2]. Briefly, total cell lysates were extracted from NPC SUNE1 cells using Nonidet P-40 (NP-40) lysis buffer. 1.2 mg total proteins were incubated with 30μl Protein A/G-Sepharose™ 4B for 4 h at 4°C, followed by centrifugation for 5 min at 4 °C. The clarified supernatants were incubated with 2 μg anti-EphA2 antibody and 30μl Protein A/G-Sepharose 4B overnight at 4 °C, followed by centrifugation for 5 min 4 °C. The Sepharose 4B beads were washed 3 times with PBS at 4 °C, and boiled in 2×SDS-PAGE loading buffer for 5 min to elute protein complexes. The elutants were separated on SDS-PAGE gel, followed by coomassie brilliant blue G250 staining. About 130 kDa protein bands were excised from the gels, in-gel trypsin digested, and then performed LC-MS/MS methylated peptide sequencing analysis. Before analysis on the Q Exactive (ThermoFisher Scientific, San Jose, CA, USA), peptides were separated using an Ultimate 3000 RSLCnano system.

Mass spectrometric data were processed by Proteome discoverer 1.4 (PD1.4, ThermoFisher Scientific) and Mascot. Data were searched against Uniprot-EphA2 (human) database (downloaded from www.uniprot.org) concatenated with reverse decoy database. The maximum missed cleavages sites was set to a default value of 2 and enzyme specificity was trypsin (full). Precursor mass tolerance was set to 5 ppm and fragments mass tolerance was set to 0.02 Da. Dynamic modification set as Oxidized, methyl (R/K), Dimethyl (R/K) and static modification set as carbamidomethyl. The false discovery rate (FDR) was adjusted to < =1% at protein, peptide and peptide-spectrum match (PSM) levels. Unique peptide values ≥1 indicated identity or extensive homology ($P < 0.05$) and were considered significant.

Duolink proximity ligation assay (PLA)

Duolink *In Situ* Red Starter Kit Mouse/Rabbit kit was used to detect PRMT5 and

EphA2 interaction as previously described by us [3]. Briefly, cells were cultured in chamber slides (Millipore), fixed in 4% paraformaldehyde for 10 min, then blocked with Duolink blocking solution and incubated with rabbit anti-PRMT5 antibody (1:200 dilution) and/or mouse anti-EphA2 antibody (1:100 dilution) overnight at 4 ℃. The cells were incubated with secondary antibodies conjugated with oligonucleotide PLA probes (anti-mouse MINUS and anti-rabbit PLUS) at 37 ℃ for 1 h. Finally, the cells were incubated with ligase at 37 ℃ for 30 min, followed by incubation with polymerase at 37 ℃ for 100 min. Nuclei were counterstained with DAPI. Images were captured using an inverted confocal fluorescent microscope (LEICA TCS SP8). The PLA signals were recognized as red fluorescent spots.

GST pull-down assay

The GST pull-down assay was performed to detect the direct interaction between PRMT5 and EphA2 as described previously by us [3]. Briefly, pET-28α expressing human PRMT5 with a Histidine (His) tag and pGEX-4T-1 expressing EphA2 with a GST tag and were transfected into *E. coli* Rosetta (DE3) (DLC204, Tsingke biotechnology), respectively. His-PRMT5 fusion protein was purified using His-Tag Protein Purification Kit (20751ES10, Yeasen), and GST-EphA2 fusion protein was purified with a GST-tag protein purification kit (P2262, Beyotime) according to the manufacturer’s instructions. 10 μg of GST or GST-EphA2 fusion protein was immobilized in 100 μL glutathione agarose and equilibrated at 4°C for 4 h. 10 μg His-tagged PRMT5 fusion protein was added to 10 μg GST-PD-L1 or GST immobilized on glutathione agarose and incubated in GST pull-down buffer at 4°C overnight. After washing with PBS buffer 5 times, the bound proteins were dissolved in 2×SDS loading buffer, separated by SDS-PAGE, and subjected to immunoblotting with antibody against GST or His.

***In vitro* methylation analysis**

The methylation level of purified EphA2 protein was detected by *in vitro* methylation analysis as previously described [4]. In brief, pGEX-4T-1 expressing EphA2 with a GST tag and were transfected into *E. coli* Rosetta (DE3), and GST-EphA2 fusion protein was purified with a GST-tag protein purification kit. PRMT5 protein or Flag-PRMT5-R368A was obtained by immunoprecipitation with PRMT5 antibody from HEK293 cells transfected with the plasmid expressing Flag-PRMT5 or Flag-PRMT5-R368A. GST-EphA2 fusion protein and immunoprecipitated PRMT5 or PRMT5-R368A protein were incubated in reaction buffer (5 mM $MgCl_2$, 50 mM Tris-HCl, 4 mM DTT, pH 8.5) containing 1 μM methyl group donor SAM (PerkinElmer, NET155V250UC) at 37 ℃ for 75 min. The reaction was stopped with SDS sample buffer, and subjected to SDS-PAGE and immunoblotting with antibody against symmetric di-methylarginine or EphA2.

Biotin pull-down assay

The interaction of peptide and EphA2 was detected using biotin pull-down assay as described previously by us [5]. In brief, 1 mg cell proteins were incubated with 30 μL streptavidin agarose beads for 4 h at 4°C, followed by centrifugation for 5 min at 4 °C. The clarified supernatants were incubated with 0-60 nM biotin-labeled PRMT5-derived P20 peptide overnight at 4℃, followed by incubation with 30 μL streptavidin agarose beads for 4 h at 4 ℃. After washing with PBS buffer 5 times, beads were boiled in 2×SDS-PAGE loading buffer for 5 min, and subjected to SDS-PAGE and immunoblotting with EphA2 antibody.

Quantitative Real-time polymerase chain reaction (qRT-PCR)

The mRNA expression of PRMT5 and EphA2 was detected using qRT-PCR in the indicated cells as described previously by us [1]. The primers are shown in the Table

S7. Briefly, total RNA was extracted from the indicated cells using Trizol reagent. Subsequently, 2 μg total RNA was reversely transcribed into cDNA using the reverse transcription (RT) kit and Oligo dT primer according to the manufacturer's instruction. The RT products were subjected to amplification through real-time PCR using QuantiFast SYBR Green PCR kit, and GAPDH was used to normalize the expression level of PRMT5 and EphA2 gene. Quantitative real-time PCR was performed on the ABI Gene Amp PCR System 9700 (ABI).

Single-cell suspension preparation

Single-cell suspensions were prepared from the xenografts. In brief, tumors were cut into pieces and subjected to digestion in a solution comprising 2% collagenase VI, 1% hyaluronidase, and 0.5% deoxyribonuclease I (DNase I) at 37 °C for 90 min. Single-cell suspensions were obtained by filtration using a 70-μm cell strainer (258368, NEST Biotechnology). After Fc receptor blocking with Human BD Fc Block™, dead cells were eliminated using the Zombie Aqua™ Fixable Viability Kit. The cells were stained with PerCP/Cyanine5.5-conjugated anti-mouse H-2K^d antibody for 30 min on ice in the dark to identify and exclude mouse-derived cells. Prepared single-cell suspensions underwent flow cytometric analysis of aldehyde dehydrogenase (ALDH) activity and CD33 expression.

Analysis of ALDH positive cell population by flow cytometry

Flow cytometry was used to analyze aldehyde dehydrogenase (ALDH) positive cell population using the Aldefluor assay kit according to the manufacturer's instruction. In Brief, 2×10^6 cells were re-suspended in 1 mL Aldefluor assay buffer, then 5 μL ALDH substrate was added to this cell suspension. After gentle mixing, 500 μL of the cell suspension was quickly transferred to a tube containing 5 μL 4-diethylaminobenzaldehyde (DEAB) reagent, an ALDH inhibitor. Both groups of

cells, 500 μL cell suspension each group, were incubated at 37 ℃ for 30 min in the dark. The cells were washed twice with cold assay buffer and centrifuged at 2000 rpm for 5 min at 4 ℃. Finally, the cells were analyzed using a FACSCanto™ II flow cytometer (BD Biosciences), and data analysis was conducted with FlowJo software, version 10.0.

Analysis of CD133 positive cell population by flow cytometry

CD133 positive cell population was analyzed by flow cytometry. Briefly, 1×10^6 cells were re-suspended in PBS containing 5% fetal bovine serum (FBS) and blocked with 5% bovine serum albumin at 4℃ for 30 min. The cells were incubated with APC-conjugated anti-CD133 antibody (1:100 dilution) or APC-conjugated rabbit isotype control IgG (1:50 dilution) 4℃ for 30 min in the dark, then the cells were washed two times with cold PBS containing 5% FBS and centrifuged at 2000 rpm for 5 min at 4 °C. Finally, the cells were analyzed using a Dxp Athena™ flow cytometer (Cytek), and data analysis was conducted with FlowJo software, version 10.0.

Immunofluorescent staining

The subcellular location of EphA2 and Cbl proteins in the indicated cells was detected using immunofluorescent staining as described previously by us [5]. In brief, cells were cultured in chamber slides (Millipore), then fixed in 4% paraformaldehyde. After permeabilization, the cells incubated with 1:300 dilution of mouse anti-EphA2 antibody or 1:200 dilution of rabbit anti-Cbl antibody. Finally, the cells were incubated with DyLight® 488 anti-mouse IgG and DyLight® 594 anti-Rabbit IgG, nuclei were counterstained with DAPI, images were captured using an inverted confocal fluorescent microscope (LEICA TCS SP8).

Immunohistochemistry and staining evaluation

The expression levels of PRMT5, EphA2, c-Myc, ALDH1A1, Nanog and Sox-2 in the formalin-fixed and paraffin-embedded tissues were detected using immunohistochemistry as described previously by us [1]. In brief, the tissue sections underwent deparaffinization using xylene, and were rehydrated through graded alcohol. Antigen retrieval was performed with 10 mmol/L sodium citrate buffer (pH 6.0). To block endogenous peroxidase activity, the sections were soaked in 3% hydrogen peroxide for 10 min. To block nonspecific antibody binding, the sections were pre-incubated with 10% nonimmune goat serum at room temperature (RT) for 15 min, followed by incubation with primary antibody overnight at 4 ℃. The sections were then incubated with biotinylated secondary antibody, followed by avidin-biotin peroxidase complex incubation at room temperature (RT) for 30 min, and stained with DAB(3,3-diaminobenzidine). Finally, tissue sections were counterstained with hematoxylin. In negative controls, a normal mouse or rabbit IgG was used in place of the primary antibodies.

Two independent pathologists, who were unaware of the clinicopathological details, evaluated and scored the immunohistochemical staining. Any disagreements were resolved through consensus. Positive reactions were defined as brown signals appeared in the cells. The intensity of staining was classified into categories: no staining as 0, weak as 1, moderate as 2, and strong as 3. The proportion of stained cells (examined in at least 500 cells) was categorized as 0 for no staining, 1 for less than 30% stained cells, 2 for 30-60% stained cells, and 3 for more than 60% stained cells. The staining score for each tissue, ranging from 0 to 6, was determined by summing the area and intensity scores. A combined staining score of ≤3 was considered to be low expression, and > 3 was considered to be high expression.

Molecular docking

Molecular docking was performed to identify the amino acid residues of Cbl and EphA2 interaction. Briefly, the docking model of Cbl-EphA2 complex was established using the ClusPro web server for protein-protein docking [6] based on the crystal structures of both Cbl (PDB ID: 2Y1M) and EphA2 (PDB ID: 1MQB). Structural illustrations were generated using the PyMOL Molecular Graphic Systems (version 0.99, Schrödinger LLC; http://www.pymol.org/).

Molecular docking was also performed to screen the protein-derived peptide disturbing PRMT5-EphA2 interaction. Briefly, the docking model of PRMT5-EphA2 complex was generated using the HDOCK web server for protein-protein docking [7] based on the crystal structures of PRMT5 (PDB ID: 4GQB) and EphA2 (PDB ID: 1MQB). Experimentally identified interaction sequences of PRMT5 and EphA2 were constrained as interaction regions during the docking process. The docking procedure generated ten potential complexes, and the most possible protein interaction mode was determined based on an analysis of residue interactions, from which the protein-derived peptide disturbing PRMT5 and-EphA2 interaction was designed.

References

1. Li JY, Xiao T, Yi HM, et al. S897 phosphorylation of EphA2 isindispensable for EphA2-dependent nasopharyngeal carcinoma cell invasion, metastasis and stem properties. *Cancer Lett*. 2019; 444:162-74.
2. Sylvestersen KB, Horn H, Jungmichel S, Jensen LJ, Nielsen ML. Proteomic analysis of arginine methylation sites in human cells reveals dynamic regulation during transcriptional arrest. *Mol Cell Proteomics*. 2014; 13(8):2072-2088.
3. Yu ZZ, Liu YY, Zhu W, et al. ANXA1-derived peptide for targeting PD-L1 degradation inhibits tumor immune evasion in multiple cancers. *J Immunother*

Cancer. 2023; 11(3): e006345.

4. Wang X, Qiu T, Wu Y, et al. Arginine methyltransferase PRMT5 methylates and stabilizes KLF5 via decreasing its phosphorylation and ubiquitination to promote basal-like breast cancer. *Cell Death Differ*. 2021; 28(10):2931-2945.
5. Feng J, Lu SS, Xiao T, et al. ANXA1 binds and stabilizes EphA2 to promote nasopharyngeal carcinoma growth and metastasis. *Cancer Res*. 2020; 80(20):4386-4398.
6. Kozakov D, Hall DR, Xia B, et al. The ClusPro web server for protein-protein docking. *Nat Protoc*. 2017; 12(2):255-278.
7. Yan Y, Tao H, He J, Huang SY. The HDOCK server for integrated protein-protein docking. *Nat Protoc*. 2020; 15(5):1829-1852.

Table S1. Tumor-initiation capacity of NPC cell lines with PRMT5 knockdown or PRMT5 knockdown and EphA2 overexpression

Cell No.		Tumor incidence of SUNE1 cells						Tumor incidence of HK1 cells					
		Days after injection						Days after injection					
		**10**	**15**	**20**	**25**	**30**	**35**	**10**	**15**	**20**	**25**	**30**	**35**
1×10^3	shCtrl	0/8	0/8	0/8	1/8	1/8	2/8	0/8	0/8	0/8	0/8	0/8	1/8
	shPRMT5	0/8	0/8	0/8	0/8	0/8	0/8	0/8	0/8	0/8	0/8	0/8	0/8
	shPRMT5 + EphA2-OE	0/8	0/8	0/8	0/8	1/8	2/8	0/8	0/8	0/8	0/8	0/8	1/8
1×10^4	shCtrl	0/8	2/8	4/8	4/8	4/8	4/8	0/8	1/8	2/8	3/8	3/8	3/8
	shPRMT5	0/8	0/8	0/8	0/8	1/8	2/8	0/8	0/8	0/8	0/8	0/8	0/8
	shPRMT5 + EphA2-OE	0/8	2/8	3/8	4/8	4/8	4/8	0/8	0/8	1/8	2/8	3/8	3/8
1×10^5	shCtrl	3/8	5/8	6/8	6/8	6/8	6/8	2/8	3/8	5/8	5/8	5/8	5/8
	shPRMT5	0/8	2/8	3/8	4/8	4/8	4/8	0/8	1/8	2/8	3/8	3/8	3/8
	shPRMT5 + EphA2-OE	3/8	4/8	6/8	6/8	6/8	6/8	1/8	2/8	4/8	5/8	5/8	5/8

Table S2. Tumor-initiation capacity of NPC cell lines with stable expression of exogenous WT EphA2 or EphA2-R816K

Cell No.		Tumor incidence of SUNE1 cells						Tumor incidence of HK1 cells					
		Days after injection						Days after injection					
		**10**	**15**	**20**	**25**	**30**	**35**	**10**	**15**	**20**	**25**	**30**	**35**
1×10^3	Vector	0/8	0/8	0/8	0/8	0/8	0/8	0/8	0/8	0/8	0/8	0/8	0/8
	EphA2-WT	0/8	0/8	0/8	1/8	2/8	2/8	0/8	0/8	0/8	0/8	1/8	1/8
	EphA2-R816K	0/8	0/8	0/8	0/8	0/8	0/8	0/8	0/8	0/8	0/8	0/8	0/8
1×10^4	Vector	0/8	0/8	0/8	1/8	2/8	2/8	0/8	0/8	0/8	0/8	0/8	0/8
	EphA2-WT	0/8	2/8	3/8	4/8	4/8	4/8	0/8	1/8	2/8	3/8	3/8	3/8
	EphA2-R816K	0/8	0/8	0/8	1/8	2/8	2/8	0/8	0/8	0/8	0/8	0/8	0/8
1×10^5	Vector	0/8	1/8	3/8	4/8	4/8	4/8	0/8	1/8	2/8	3/8	3/8	3/8
	EphA2-WT	3/8	4/8	6/8	6/8	6/8	6/8	2/8	3/8	4/8	5/8	5/8	5/8
	EphA2-R816K	0/8	2/8	3/8	4/8	4/8	4/8	0/8	1/8	2/8	3/8	3/8	3/8

Table S3. Tumor-initiation capacity of the NPC cell lines in tumor-bearing mice received P20 treatment

Cell No.		Tumor incidence of SUNE1 cells						Tumor incidence of HK1 cells					
		Days after injection						Days after injection					
		**10**	**15**	**20**	**25**	**30**	**35**	**10**	**15**	**20**	**25**	**30**	**35**
1×10^3	PBS	0/8	0/8	0/8	1/8	2/8	2/8	0/8	0/8	0/8	0/8	1/8	1/8
	P20	0/8	0/8	0/8	0/8	0/8	0/8	0/8	0/8	0/8	0/8	0/8	0/8
1×10^4	PBS	0/8	2/8	3/8	4/8	4/8	4/8	0/8	1/8	2/8	3/8	3/8	3/8
	P20	0/8	0/8	0/8	1/8	2/8	2/8	0/8	0/8	0/8	0/8	0/8	0/8
1×10^5	PBS	3/8	4/8	6/8	6/8	6/8	6/8	2/8	3/8	4/8	5/8	5/8	5/8
	P20	0/8	1/8	3/8	4/8	4/8	4/8	0/8	1/8	2/8	3/8	3/8	3/8

Table S4. Correlations between the two proteins expression and clinicopathological characteristics in NPC (n=152)

Variables	N	PRMT5			EphA2		
		Low	High	*p*	Low	High	*p*
**Age (y)**				0.431			0.094
<45	60	26	34		18	42	
≥45	92	34	58		40	52	
**Gender**				0.310			0.488
Male	105	43	62		42	63	
Female	47	17	30		16	31	
**Histologic type (WHO)**				0.220			0.342
Non-keratinized type							
Differentiation	42	19	23		18	24	
Undifferentiation	110	38	72		36	74	
**Primary tumor(T) stage**				0.052			0.078
T1-2	74	35	39		32	42	
T3-4	78	27	57		23	55	
**Lymph node(N) metastasis**				0.020			0.033
N0	75	36	39		35	40	
N1-3	77	24	53		23	54	
**Distant metastasis(M)**				0.001			0.001
M0	101	51	50		50	51	
M1	51	9	42		8	43	
**Clinical TNM stage**				0.000			0.000
I-II	66	40	26		36	30	
III-IV	86	20	66		22	64	
**Smoking history**				0.149			0.159
Yes	60	18	39		18	39	
No	124	42	53		40	55	
**Recurrence**				0.000			0.000
Yes	69	16	53		18	51	
No	83	44	39		40	43	

Chi-square test, $P < 0.05$ was considered statistically significant.

Table S5. Univariate and cox multivariate analyses of prognostic factors for disease-free survival (n=152)

Variables	Disease free survival (DFS)				Overall survival (OS)			
	Univariate analysis		Multivariate analysis		Univariate analysis		Multivariate analysis	
	P	**HR (95% CI)**	***P***	**HR (95% CI)**	***P***	**HR (95% CI)**	***P***	**HR (95% CI)**
**Age (y)**								
<45 vs. ≥45	0.067	0.560(0.335-0.937)	0.083	0.712(0.358-1.203)	0.092	0.433(0.230-0.814)	0.144	0.387(0.207-0.985)
**Gender**								
Male vs. Female	0.269	1.335(0.797-2.323)	0.524	1.796(0.752-2.799)	0.813	1.075(0.589-1.965)	0.908	1.822(1.201-3.379)
**Histologic type (WHO)**								
Differentiation vs. Undifferentiation	0.069	0.569 (0.368-1.457)	0.107	0.808(0.453-0.997)	0.074	0.792(0.525-1.237)	0.249	0.785(0.594-1.982)
**Primary tumor(T) stage**								
T1-2 vs. T3-4	0.058	0.387 (0.133-0.711)	0.089	0.763(0.686-1.529)	0.091	0.635(0.428-1.254)	0.167	0.745(0.479-1.605)
**Lymph node(N) metastasis**								
N0 vs. N1-3	0.002	0.463(0.283-0.756)	0.010	0.504(0.285-0.873)	<0.001	0.276(0.147-0.520)	0.002	0.695(0.579-0.934)
**Distant metastasis(M)**								
M0 vs. M1	<0.001	0.393(0.244-0.631)	0.001	0.387(0.248-0.899)	<0.001	0.283(0.162-0.493)	0.001	0.359(0.208-0.684)
**Clinical TNM stage**								
I-II vs. III-IV	0.001	0.426(0.254-0.713)	0.030	0.587(0.265-0.697)	<0.001	0.282(0.144-0.551)	0.002	0.583(0.329-0.799)
**Smoking history**								
Yes vs. No	0.244	0.349(0.217-0.564)	0.674	0.557(0.375-0.807)	0.151	0.667(0.384-1.159)	0.452	0.737(0.528-0.982)
**PRMT5 level**								
High vs. Low	<0.001	0.363(0.211-0.624)	<0.001	0.347(0.189-0.484)	<0.001	0.344(0.179-0.660)	<0.001	0.237(0.101-0.693)
**EphA2 level**								
High vs. Low	<0.001	0.297(0.169-0.522)	<0.001	0.478(0.293-0.735)	<0.001	0.326(0.170-0.626)	<0.001	0.382(0.105-0.749)
**PRMT5/EphA2 level**								
High/High vs. High/Low and Low/High	<0.001	0.234(0.120-0.456)	<0.001	0.398(0.148-0.878)	<0.001	0.253(0.116-0.549)	<0.001	0.356(0.273-0.742)

Table S6. The clinicopathological characteristics of 152 patients with NPC

Variables	No. of patients (n)	Percent
**Age (y)**		
<45	60	39.5
≥45	92	60.5
**Gender**		
Male	105	69.1
Female	47	30.9
**Histologic type (WHO)**		
Non-keratinized type		
Differentiation	42	27.6
Undifferentiation	110	72.4
**Primary tumor(T) stage**		
T1-2	74	48.7
T3-4	78	51.3
**Lymph node(N) metastasis**		
N0-1	75	49.3
N2-3	77	50.7
**Distant metastasis(M)**		
M0	101	66.4
M1	51	33.6
**Clinical TNM stage**		
I-II	66	43.4
III-IV	86	56.6
**Smoking history**		
Yes	60	39.5
No	124	60.5
**Recurrence**		
Yes	69	45.4
No	83	54.6

Table S7. The primers used for the amplification of the genes by qRT-PCR

No.	Gene name	GenBank Accession No	Primer sequence
1	Human PRMT5	NM_006109	F: CCTGTGGAGGTGAACACAGT<br>R: AGAGGATGGGAAACCATGAG
2	Human EphA2	NM_004431	F: TGGCTCACACACCCGTATG<br>R: GTCGCCAGACATCACGTTG
3	Human GAPDH	NM_002046	F: CAGGAGGCATTGCTGATGAT<br>R: GAAGGCTGGGGCTCATTT

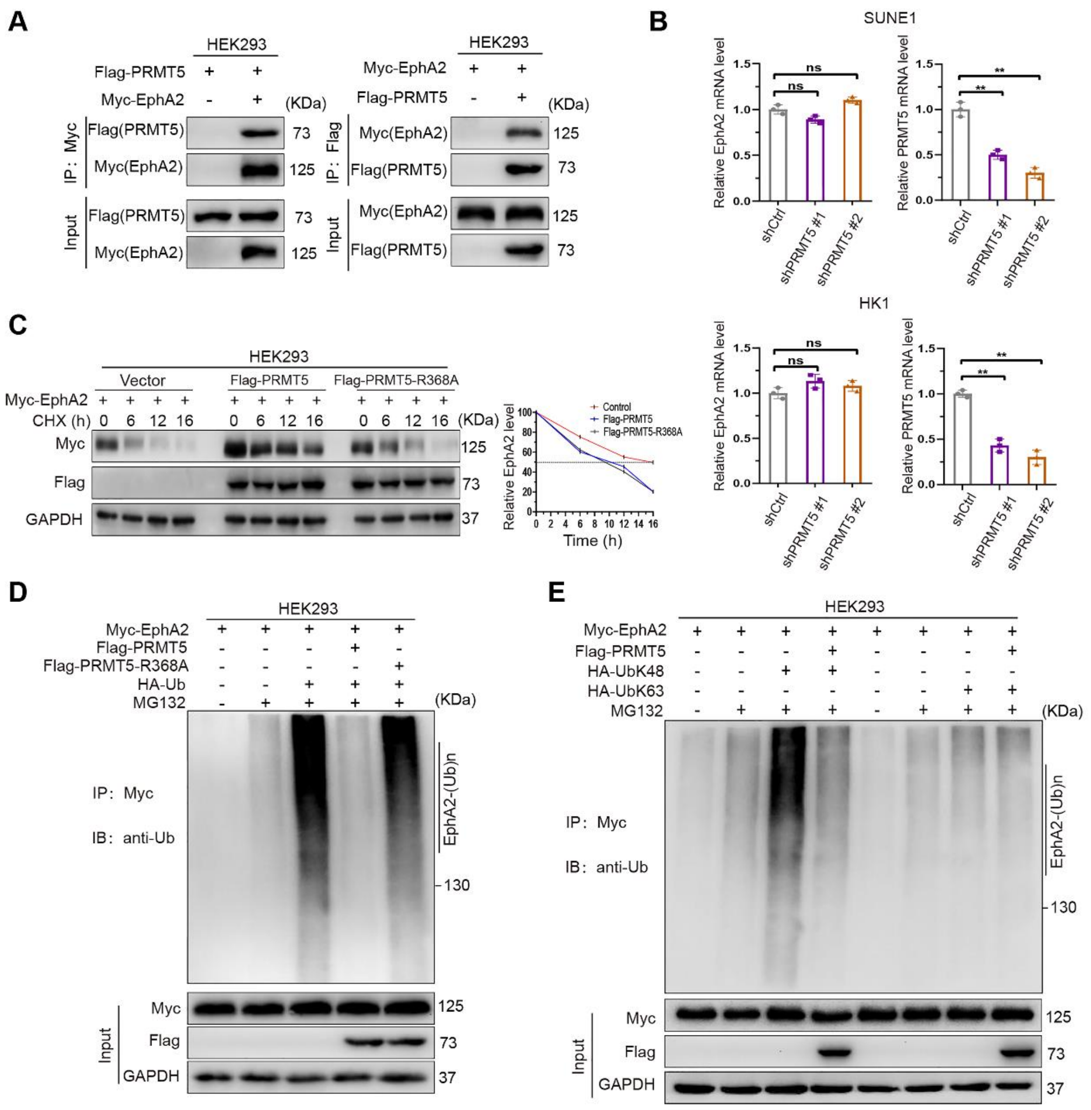

Figure S1. PRMT5 binds and stabilizes EphA2 protein via inhibiting its ubiquitination and degradation. (**A**) Co-Immunoprecipitation (Co-IP) showing PRMT5-EphA2 interaction in the HEK293 cells ectopically expressing the Flag-tagged PRMT5 and Myc-tagged EphA2. (**B**) qRT-PCR showing the levels of EphA2 mRNA in the PRMT5 knockdown SUNE1 and HK1 NPC cells and their shCtrl control cells. Numbers represent means ± SD. ***P*< 0.01; ns, no significance. (**C**) Western blot showing that WT PRMT5 but not catalytically inactive mutant PRMT5-R368A increased EphA2 protein stability in the HEK293 cells treated with 20 μg/mL CHX for indicated times. (**D**) Co-IP showing that WT PRMT5 but not PRMT5-R368A decreased EphA2 polyubiquitination in the HEK293 cells transfected with indicated plasmids. (**E**)

Co-IP showing the type of PRMT5-inhibited EphA2 polyubiquitination. Total cell proteins from HEK293 cells transfected with indicated plasmids were subjected to immunoprecipitation with anti-EphA2 antibody, followed by immunoblotting with anti-polyubiquitin antibody. shPRMT5, PRMT5 knockdown by shRNA; shCrtl, scramble nontarget shRNA; WT, wild-type; IP, Immunoprecipitation; IB, Immunoblotting; CHX, cycloheximide.

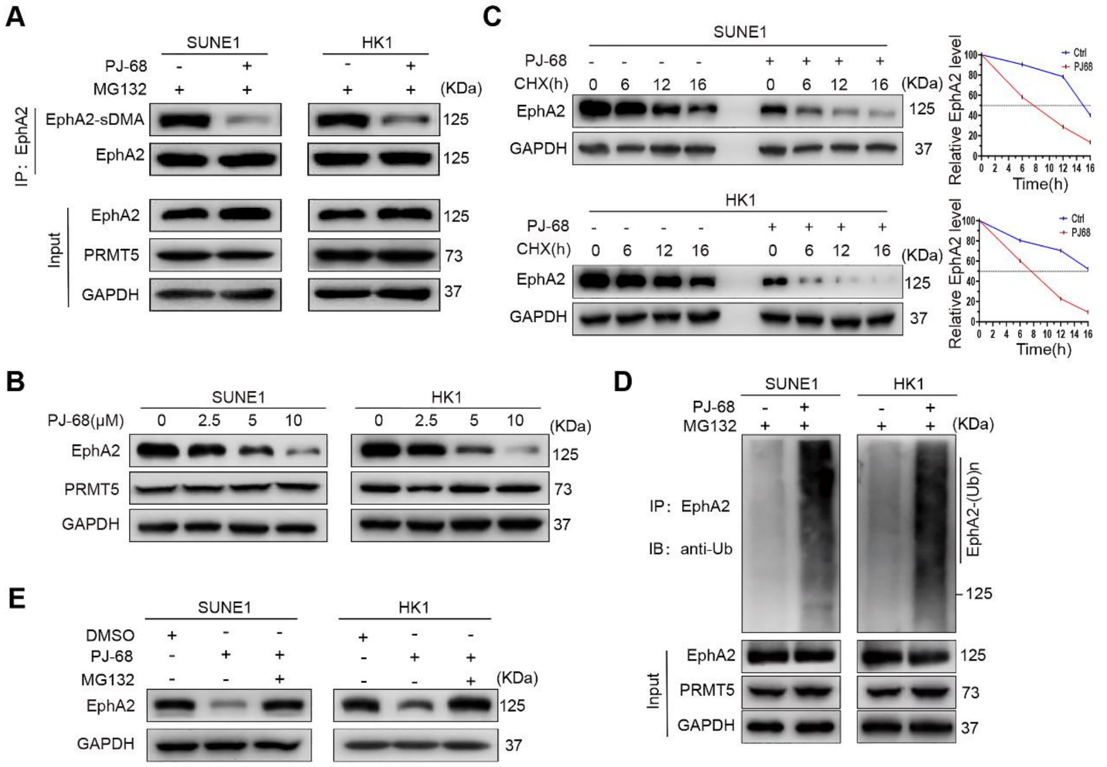

Figure S2. PRMT5 inhibitor PJ-68 decreases EphA2 stability by ubiquitin proteasome pathway in NPC cells. (**A**) Co-IP showing that PJ-68 decreased EphA2 methylation level in the SUNE1 and HK1 NPC cells. NPC cells were treated with 10 μM PJ-68 for 12 hours, and subjected to EphA2 methylation analysis. (**B**) Western blot showing that PJ-68 reduced EphA2 protein levels in the SUNE1 and HK1 NPC cells in a dose dependent manner. (**C**) Western blot showing that PJ-68 decreased EphA2 protein stability in the SUNE1 and HK1 NPC cells treated with 20 μg/mL CHX for indicated times. (**D**) Co-IP showing that PJ-68 increased EphA2 polyubiquitination

levels in the SUNE1 and HK1 NPC cells. (**E**) Western blot showing reversion of EphA2 protein levels by proteasome inhibitor MG132 in the PJ-68-treated SUNE1 and HK1 NPC cells. Cancer cells were treated with 10 μM PJ-68 for 12 hours, followed by 10 μM MG132 treatment for 12 hours. sDMA, symmetric dimethylarginine; CHX, cycloheximide; Ub, ubiquitin; IP, Immunoprecipitation; IB, Immunoblotting.

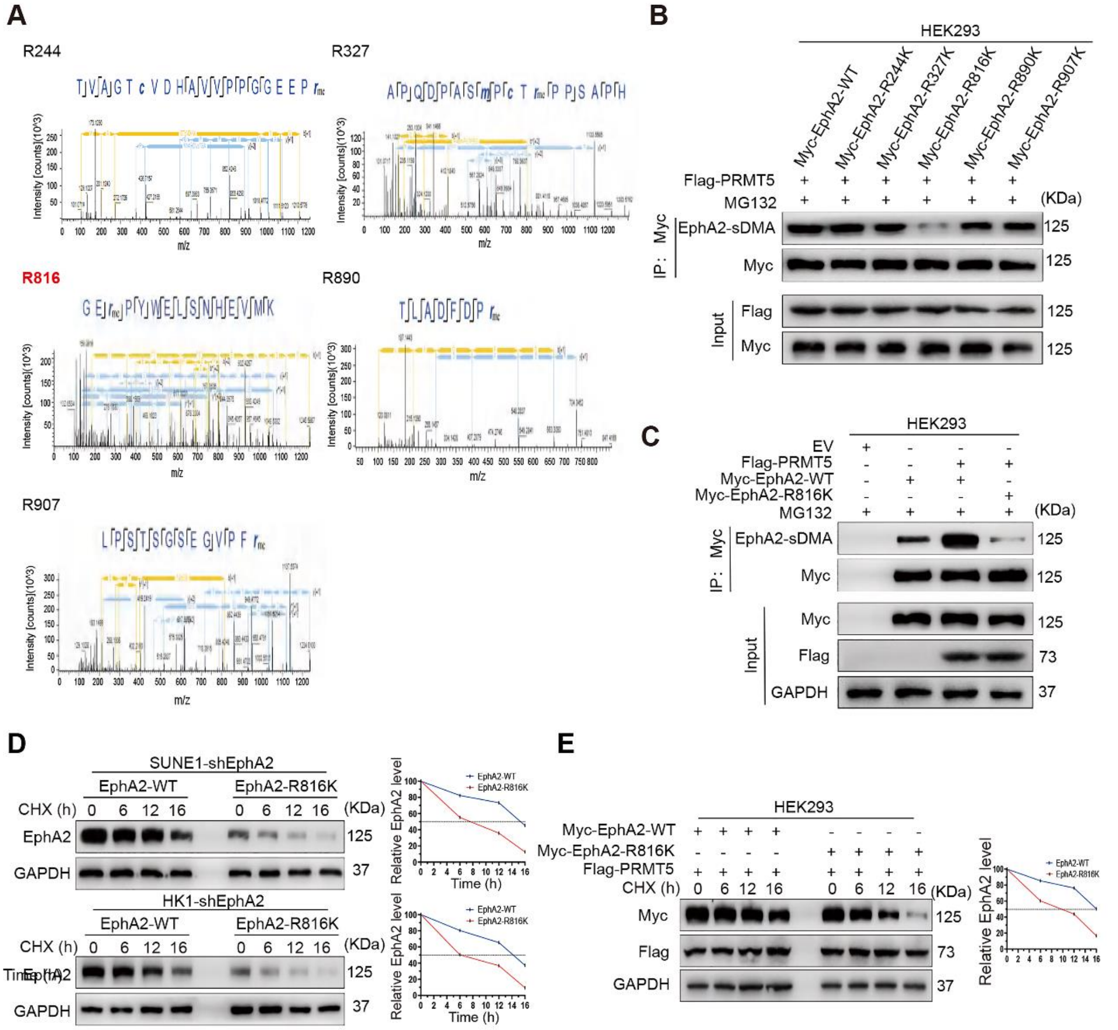

Figure S3. PRMT5 increases EphA2 protein stability by catalyzing di-methylation of EphA2 at R816. (**A**) Identification of di-methylated-arginines of EphA2 by immunoprecipitation conjugated with mass spectrometry analysis (IP-MS). Proteins from NPC HK1 cells were immunoprecipitated with anti-EphA2 antibody, resolved on SDS-PAGE and Coomassie blue stained, and then approximately 130 kDa protein bands were retrieved and analyzed by mass spectrometry (MS). Arginine 244 (R244),

R327, R816, R890 and R907 were identified as di-methylation sites of EphA2 protein. (**B**) Co-IP showing that R816K mutation but not R244K, R327K, R890K or R907K mutation decreased PRMT5-catalyzing EphA2 methylation levels in the HEK293 cells. (**C**) Co-IP showing that PRMT5 increases the methylation level of WT EphA2 but not methylation inaction mutant EphA2-R816K in the HEK293 cells transfected with indicated plasmids. (**D**) Western blot showing the protein stability of exogenous WT EphA2 and EphA2-R816K in the EphA2 knockdown NPC cells treated with 20 μg/mL CHX for indicated times. (**E**) Western blot showing the protein stability of WT EphA2 and EphA2-R816K in the HEK293 cells treated with 20 μg/mL CHX for indicated times. shEphA2, endogenous EphA2 knockdown by shRNA; sDMA, symmetric dimethylarginine; WT, wild-type; EV, empty vector; CHX, cycloheximide; IP, Immunoprecipitation.

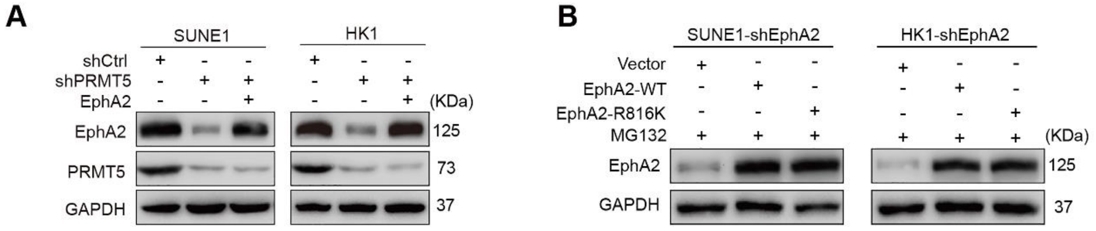

Figure S4. Establishment of stably transfected NPC cell lines. (**A**) Establishment of SUNE1 and HK1 NPC cell lines with PRMT5 knockdown, SUNE1 and HK1 NPC cell lines with PRMT5 knockdown and EphA2 overexpression and their control cell lines. (**B**) Establishment of SUNE1 and HK1 NPC cell NPC lines with stable expression of WT EphA2 or EphA2-R816K using endogenous EphA2 knockdown NPC cells and their control cell lines. shPRMT5, PRMT5 knockdown by shRNA; shCrtl, scramble nontarget shRNA; WT, wild-type.

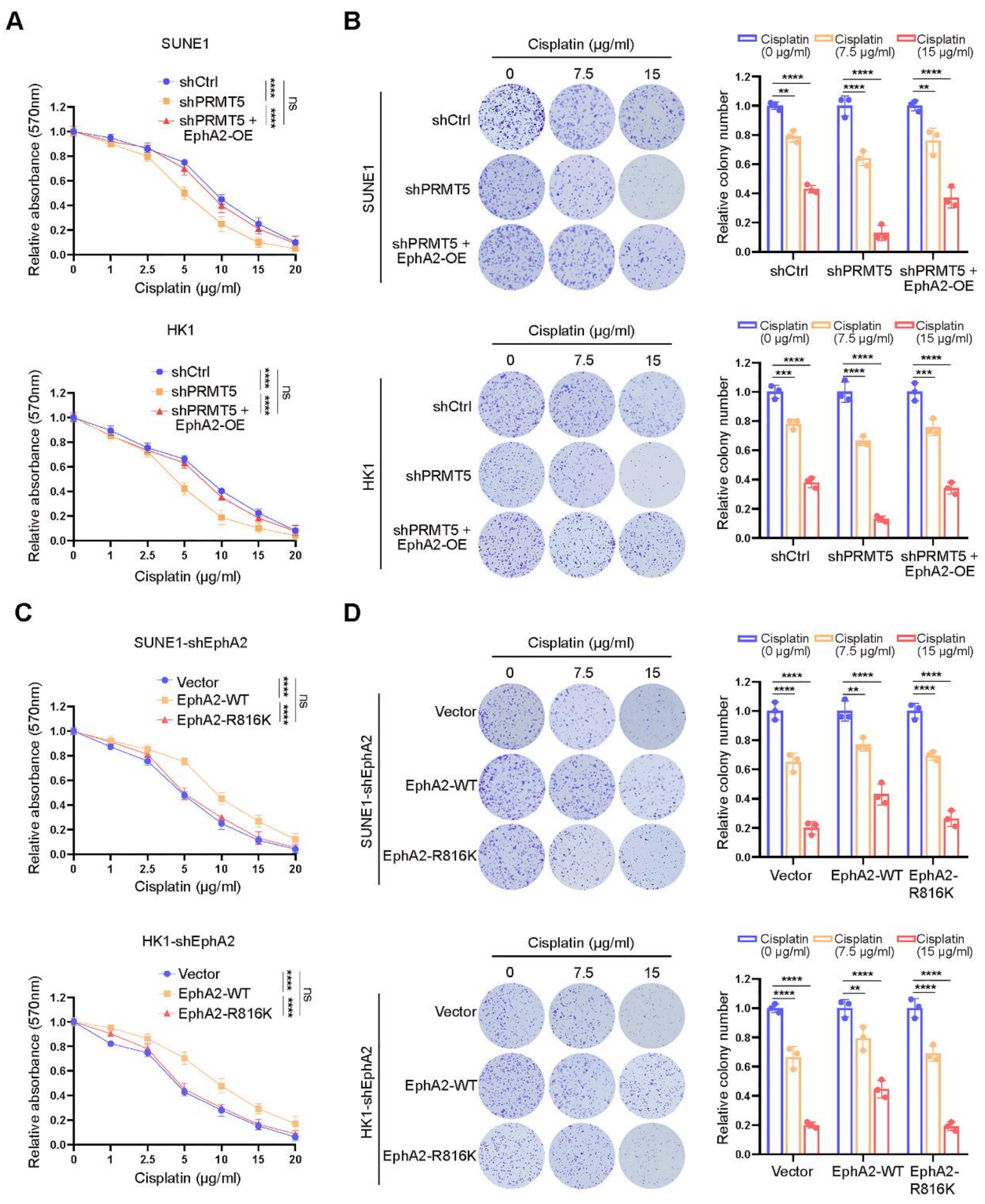

Figure S5. PRMT5 promotes NPC cell chemoresistance by methylating and stabilizing EphA2. (**A, B**) PRMT5 knockdown, PRMT5 knockdown and EphA2 overexpression, and shCtrl control NPC cells were treated with indicated concentrations of cisplatin, and cell proliferation was determined by MTT assay (**A**) and plate clone formation assay (**B**). (**C, D**) Endogenous EphA2 knockdown NPC cells with stable expression of exogenous WT EphA2 or EphA2-R816K, and vector control NPC cells were treated with indicated concentrations of cisplatin, and cell proliferation

was determined by MTT assay (**C**) and plate clone formation assay (**D**). shEphA2, endogenous EphA2 knockdown by shRNA; EphA2-OE, EphA2 overexpression; shCrtl, scramble nontarget shRNA; WT, wild-type. Numbers represent mean ± SD. **P* < 0.05; ****P* < 0.01; ****P* < 0.001; *****P* < 0.0001; ns, no significance.

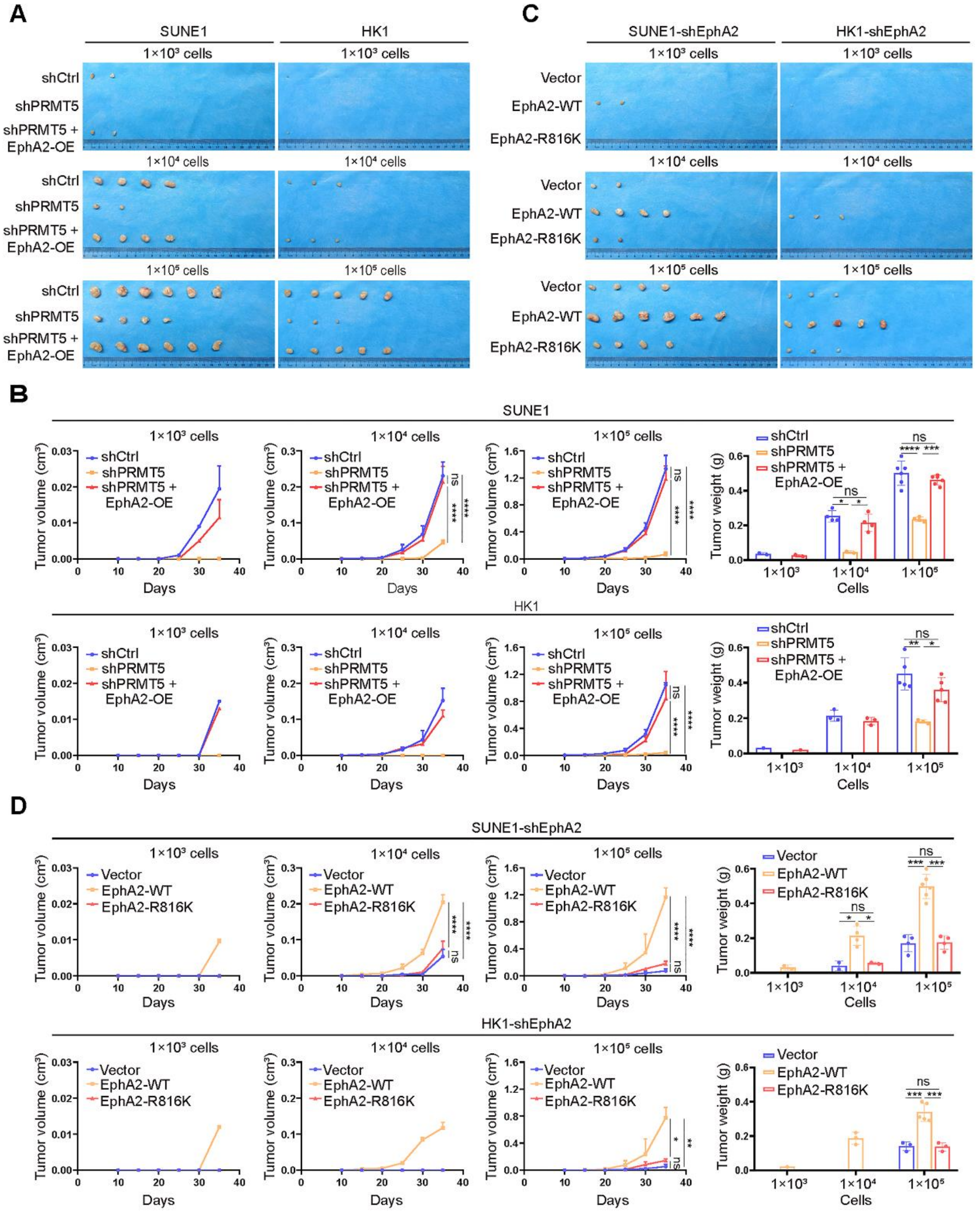

Figure S6. Tumor-initiating capacity assay showing the effect of PRMT5-methylating and -stabilizing EphA2 on *in vivo* NPC cell stemness. (**A**) The

photographs of xenografts at 5 weeks after subcutaneous implantation of PRMT5 knockdown, PRMT5 knockdown and EphA2 overexpression, and shCtrl control NPC cells into NOD-SCID mice. 8 mice per group. (**B**) Tumor growth curves (*left*) and average tumor weight (*right*) of 1×10^3, 1×10^4 and 1×10^5 RMT5 knockdown, PRMT5 knockdown and EphA2 overexpression, and shCtrl control NPC cells at 5 weeks after subcutaneous implantation. (**C**) The photographs of xenografts at 5 weeks after subcutaneous implantation of NPC cells expressing exogenous WT EphA2 or EphA2-R816K, and vector control NPC cells into NOD-SCID mice. 8 mice per group. (**D**) Tumor growth curves (*left*) and average tumor weight (*right*) of 1×10^3, 1×10^4 and 1×10^5 NPC cells expressing exogenous WT EphA2 or EphA2-R816K, and vector control NPC cells at 5 weeks after subcutaneous implantation. shEphA2, endogenous EphA2 knockdown by shRNA; shCrtl, scramble nontarget shRNA; EphA2-OE, EphA2 overexpression; WT, wild-type. Numbers represent mean ± SD. *$P < 0.05$; **$P < 0.01$; ***$P < 0.001$; ****$P < 0.0001$; ns, no significance.

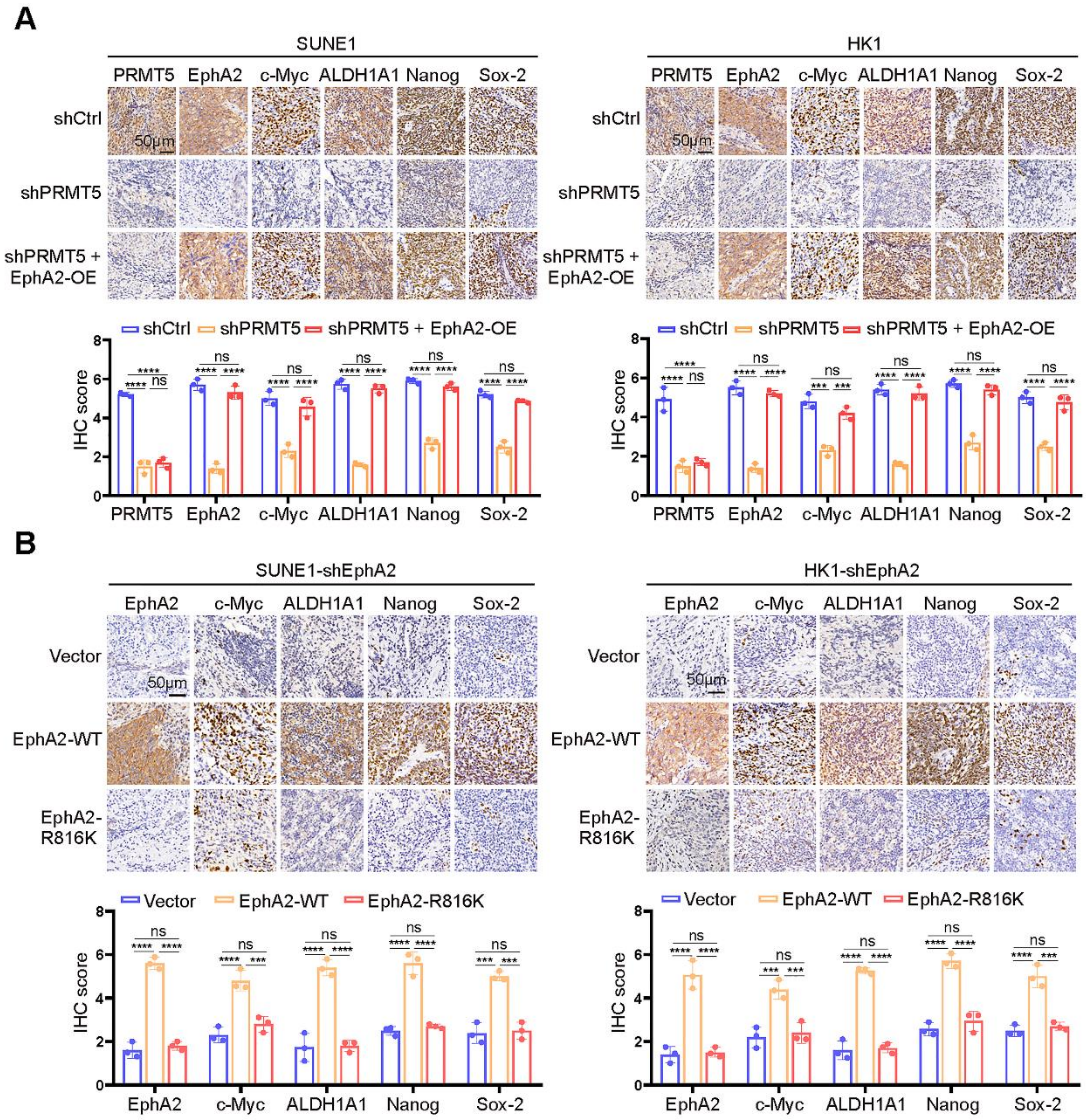

Figure S7. Immunohistochemistry (IHC) showing that the effect of PRMT5 knockdown (**A**) and methylation inactivation mutant EphA2-R816K (**B**) on the expression of EphA2, c-Myc, ALDH1A1, Nanog and Sox-2 in the xenografts. Representative IHC images are shown on the top, and statistical analysis is presented on the bottom. Scale bar, 50 μm. shEphA2, EphA2 knockdown by shRNA; shCrtl, scramble nontarget shRNA; EphA2-OE, EphA2 overexpression; WT, wild-type. Numbers represent mean ± SD. ****P* < 0.001; *****P* < 0.0001; ns, no significance.

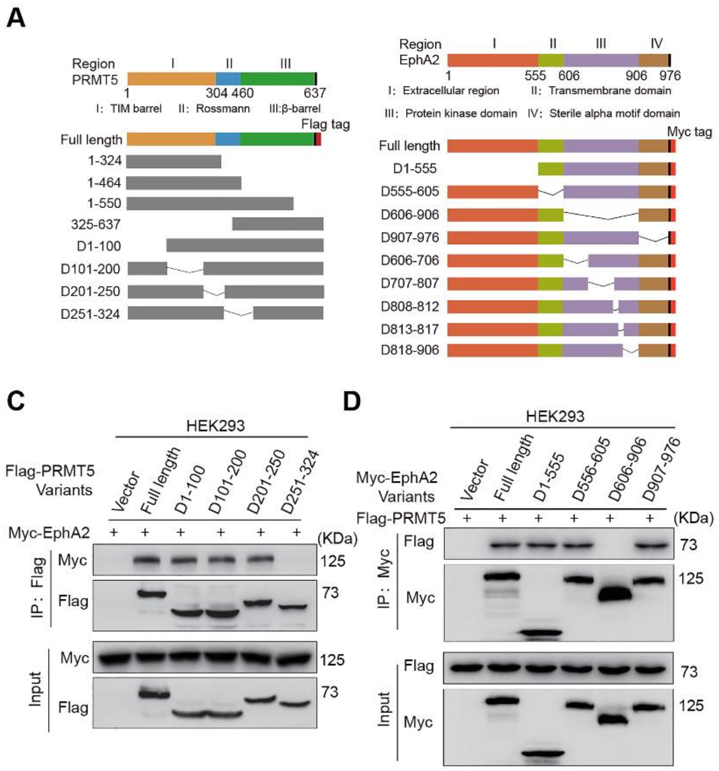

Figure S8. Mapping of the binding regions of PRMT5 and EphA2. (**A**) Diagrammatic sketch of EphA2, PRMT5 and their deleted forms. The main regions of both proteins are indicated. Numbers indicate amino acid position within the sequence. D, deletion. (**B**, **C**) Co-IP showing the region of PRMT5 bound to EphA2. Total cell proteins from HEK293 cells transfected with indicated plasmids were subjected to immunoprecipitation with anti-Flag (PRMT5) antibody, followed by immunoblotting with antibodies against Myc (EphA2) or Flag (PRMT5). (**D**, **E)** Co-IP showing the region of EphA2 bound to PRMT5. Total cell proteins from HEK293 cells transfected with indicated plasmids were subjected to immunoprecipitation with anti-Myc (EphA2) antibody, followed by immunoblotting with antibodies against Flag (PRMT5) or Myc (EphA2). IP, Immunoprecipitation.

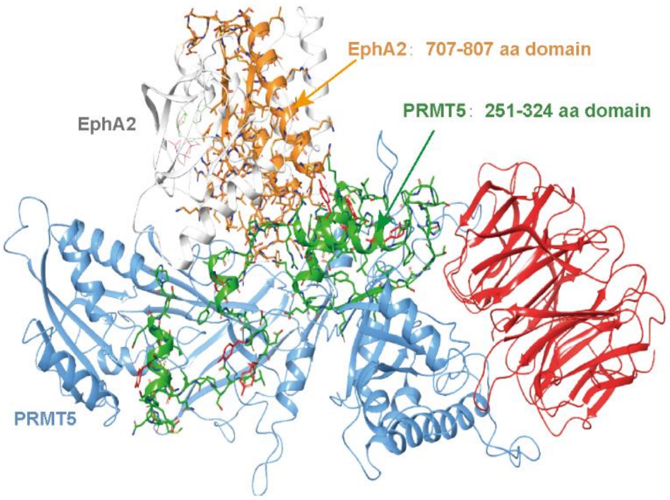

Figure S9. Molecular docking model for PRMT5-EphA2 complex. EphA2 (Protein Data Bank code: 1MQB) and PRMT5 (Protein Data Bank code: 4GQB) are colored light grey and light blue, respectively. EphA2 707-807 aa region (Reddish gold) binds to PRMT5 251-324 aa region (green). Residues within these regions are specifically depicted using a stick model. MEP50 interacting with PRMT5 is colored red. The continuous sequence SYLQYLEYLSQNRPPPNAYE (279-298aa) of PRMT5 and continuous sequence KYLANMNYVHR (728-738aa) of EphA2 were found to be involved in interactions, forming multiple interaction hotspots.

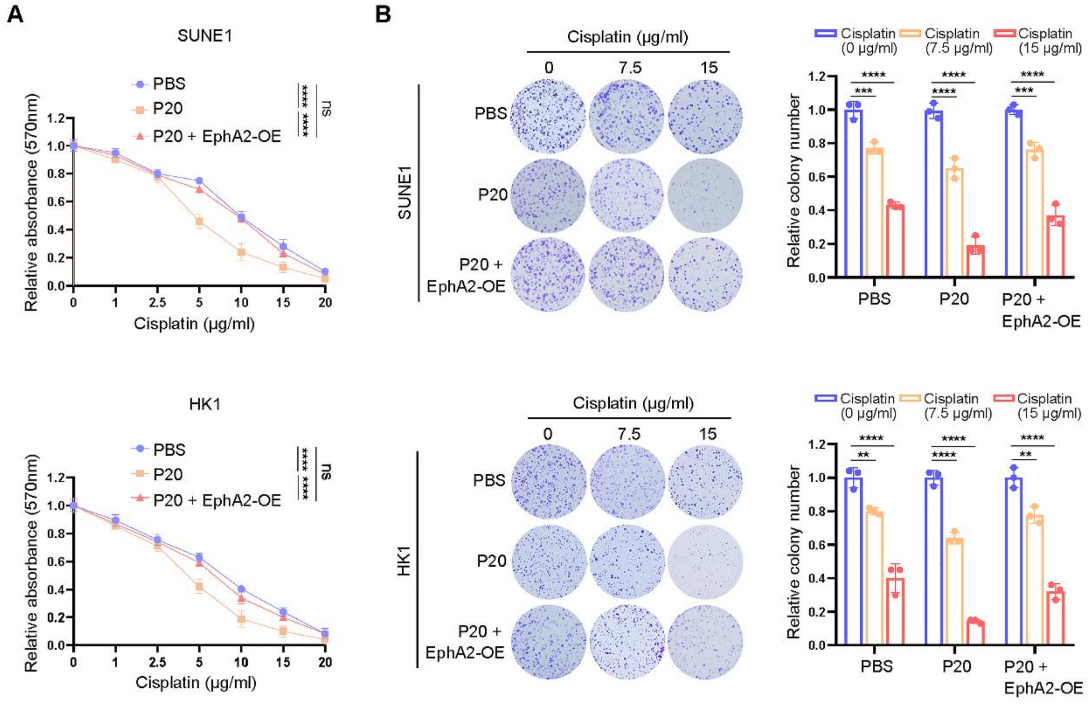

Figure S10. P20 peptide increases NPC cell chemosensitivity. SUNE1 and HK1 NPC cells and their respective EphA2 overexpression were treated with 10μM P20 or P20 in combination with the indicated concentrations of cisplatin, and cell proliferation was determined by MTT assay (**A**) and plate clone formation assay (**B**). EphA2-OE, EphA2 overexpression. Numbers represent mean ± SD. ***$P < 0.001$; ****$P < 0.0001$; ns, no significance.

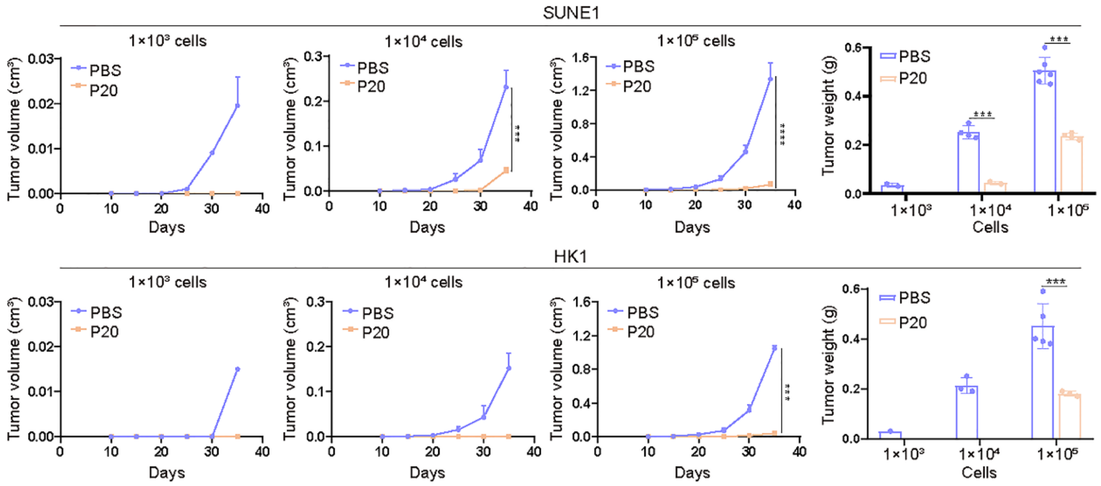

Figure S11. Tumor-initiating capacity assay showing the effect of P20 peptide on *in vivo* NPC cell stemness. Serial dilutions (1×10^3, 1×10^4 and 1×10^5) of SUNE1 and HK1 NPC cells were subcutaneously injected into NOD-SCID mice respectively (n=8 mice each group), and tumor-bearing mice received P20 treatment via peritoneal injection. Tumor volume was periodically monitored and tumor growth curves are shown on the left, and average tumor weights at 5 weeks after subcutaneous implantation are presented on the right. Peritoneal injection of PBS buffer served as a control. Numbers represent mean ± SD. ***$P < 0.001$; ****$P < 0.0001$; ns, no significance.
